# Sex Differences in Drug-Induced Arrhythmogenesis

**DOI:** 10.3389/fphys.2021.708435

**Published:** 2021-08-19

**Authors:** Mathias Peirlinck, Francisco Sahli Costabal, Ellen Kuhl

**Affiliations:** ^1^Department of Mechanical Engineering, Stanford University, Stanford, CA, United States; ^2^Department of Mechanical and Metallurgical Engineering, School of Engineering, Pontificia Universidad Católica de Chile, Santiago, Chile; ^3^Institute for Biological and Medical Engineering, Schools of Engineering, Medicine and Biological Sciences, Pontificia Universidad Católica de Chile, Santiago, Chile; ^4^Millennium Nucleus for Cardiovascular Magnetic Resonance, Santiago, Chile

**Keywords:** multiscale modeling and simulation, cardiac electrophysiology, machine learning, multi-fidelity Gaussian process classification, active learning, sex differences, arrhythmia, drugs

## Abstract

The electrical activity in the heart varies significantly between men and women and results in a sex-specific response to drugs. Recent evidence suggests that women are more than twice as likely as men to develop drug-induced arrhythmia with potentially fatal consequences. Yet, the sex-specific differences in drug-induced arrhythmogenesis remain poorly understood. Here we integrate multiscale modeling and machine learning to gain mechanistic insight into the sex-specific origin of drug-induced cardiac arrhythmia at differing drug concentrations. To quantify critical drug concentrations in male and female hearts, we identify the most important ion channels that trigger male and female arrhythmogenesis, and create and train a sex-specific multi-fidelity arrhythmogenic risk classifier. Our study reveals that sex differences in ion channel activity, tissue conductivity, and heart dimensions trigger longer QT-intervals in women than in men. We quantify the critical drug concentration for dofetilide, a high risk drug, to be seven times lower for women than for men. Our results emphasize the importance of including sex as an independent biological variable in risk assessment during drug development. Acknowledging and understanding sex differences in drug safety evaluation is critical when developing novel therapeutic treatments on a personalized basis. The general trends of this study have significant implications on the development of safe and efficacious new drugs and the prescription of existing drugs in combination with other drugs.

## 1. Introduction

It is well-established that there are important discrepancies between male and female cardiac electrophysiology. Electrocardiogram differences between men and women include a faster resting heart rate in women, a longer corrected QT interval, and a lower QT dispersion (James et al., 2007; Yarnoz and Curtis, 2008). Despite an increasing recognition, essential knowledge gaps remain in the mechanistic understanding of these sex differences, warranting further investigation (Asatryan et al., 2021). Here, to focus, we demonstrate the effect that sex differences play for one particular example, drug-induced arrhythmogenicity.

Drugs often have undesired side effects. In the heart, they can induce global changes in the electrical activity of the tissue by interacting with specific ionic channels in cardiac cells. Doing so, some compounds can induce arrhythmia known to precipitate into ventricular fibrillation and sudden cardiac death. These arrhythmia are typically associated with drugs that prolong the repolarization stage of the cardiomyocyte action potential (Po et al., 1999). Consequently, before any drug can enter the market, its pro-arrhythmic risk needs to be assessed. Currently, the gold standard for cardiac safety assessment focuses on the experimental measurement of the pharmacological block of the rapid delayed potassium rectifier current in single cell experiments (Redfern et al., 2003) and electrocardiographic analyses looking for QT prolongation in animal models or humans (Gintant et al., 2016). These biomarkers show good sensitivity but low specificity, potentially preventing useful drugs to reach the market (Sager, 2008). Moreover, these risk assessment procedures are slow and expensive to conduct. A recent initiative of regulatory agencies, drug design companies, and cardiovascular researchers suggested to address these limitations by new mechanistic assays that predict the pro-arrhythmic risk of new drugs using computational modeling (Sager et al., 2014). In response to this initiative, a collection of novel mechanistic computational paradigms for drug-induced arrhythmogenesis prediction have been proposed ranging in complexity from ventricular myocyte models (Mirams et al., 2011; Passini et al., 2017) to transmural cable simulations (Moreno et al., 2013; Romero et al., 2018), and from planar and cubic tissue slabs (Kubo et al., 2017; Yang et al., 2020; Margara et al., 2021) to ultra-high resolution, multiscale heart models (Wilhelms et al., 2012; Okada et al., 2015; Sahli Costabal et al., 2018a; Hwang et al., 2019). Over the past few years, these physics-based modeling approaches have been increasingly combined with machine learning approaches to further improve mechanistic arrhythmogenic risk classification (Lancaster and Sobie, 2016; Polak et al., 2018; Sahli Costabal et al., 2019a,c).

Even though drug-induced arrhythmogenicity has been reported to occur twice as often in women than in men (Makkar, 1993; Coker, 2008), the role that sex differences play in arrhythmogenic risk classification remains largely understudied. Current computational mechanistic risk predictors use mathematical models of cardiac cells calibrated on *in vitro* studies, that often tend to be male-dominated (Ramirez et al., 2017). As such, sex bias can be expected to propagate through these models into the actual risk stratification. Consequently, there is a strong need to study the multiscale sex differences in cardiac electrophysiology and how these discrepancies translate into sex-specific arrhythmogenic risk stratification in more detail.

**Figure 1:**
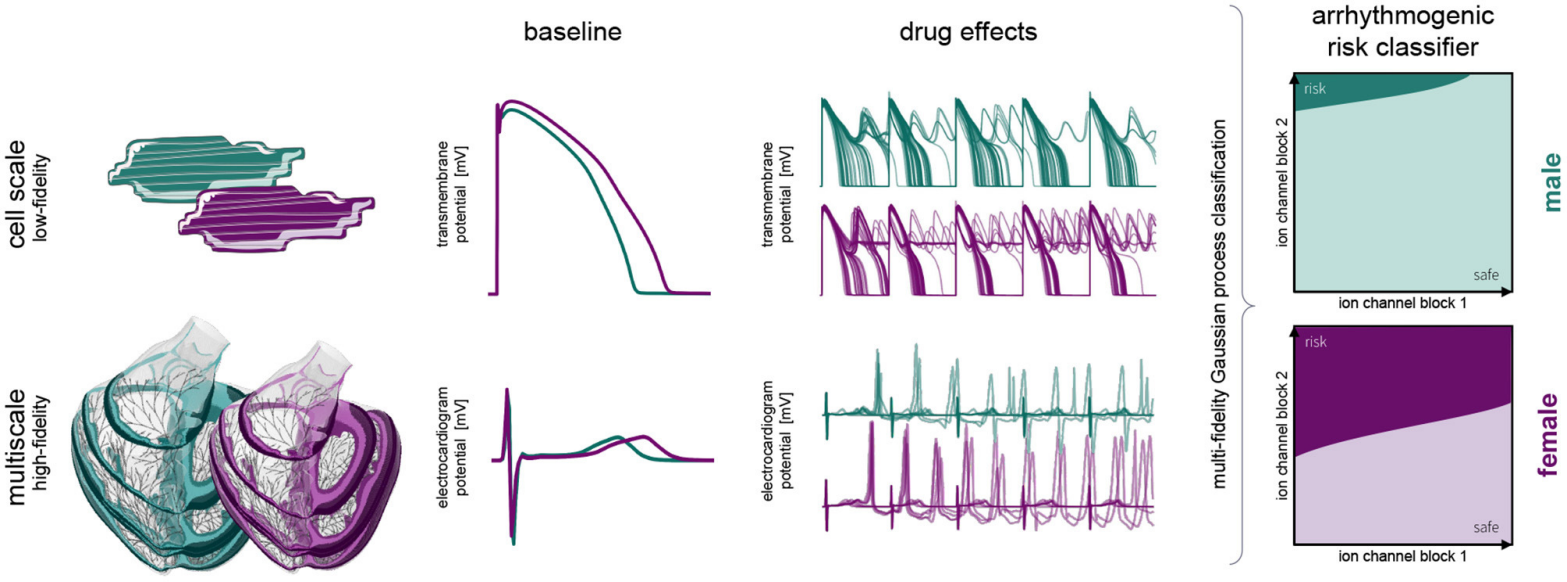
**Sex differences in drug-induced arrhythmogenesis: a combined multiscale modeling and machine learning approach**. We develop individual male and female low-fidelity cell and high-fidelity multiscale exposure-response simulators. These simulators take into account differences in subcellular ion channel activity between men and women for the low-fidelity exposure-response proxy. The high-fidelity model also takes into account sex differences tissue-level conductivity and organ-scale geometry. We perform an arrhythmic sensitivity study of the male and female heart to drug-specific ion channel blocking and susbsequently combine low-fidelity cell-scale and high-fidelity multiscale modeling to delineate arrhythmogenic risk classification boundaries for men and women.

Figure 1 provides a schematic overview of our study. Here, we build independent male and female low-fidelity cell-scale and high-fidelity multiscale cardiac electrophysiology exposure-response simulators incorporating experimentally quantified sex differences at the subcellular, cellular, tissue and organ level. Using logistic regression, we studied the pro- and anti-arrhythmic effects that drug-induced ion channel blocking has on the male and female heart individually. Combining high-performance computing and multiscale modeling with machine learning techniques, including multi-fidelity Gaussian process classification and active learning, we developed two sex-specific drug-induced multi-fidelity arrhythmogenic risk classifiers. Finally, both classifiers were used to quantify the male and female arrhythmogenic susceptibility of a high, intermediate, and low risk drug.

## 2. Materials and Methods

### 2.1. Multiscale Modeling of Cardiac Electrophysiology

We model the electrophysiological behavior of cardiac tissue using the monodomain model (Clayton et al., 2011; Sahli Costabal et al., 2018a). This model's main variable is the transmembrane potential ϕ, the difference between the intra- and extra-cellular potentials. The transmembrane potential is governed by a reaction-diffusion equation (Krishnamoorthi et al., 2014),

(1) $$\begin{array}{l} \dot{\phi }=\text{div}\left(D\cdot \nabla \phi \right)+f^{\phi }, \end{array}$$

Here, we introduce the source term *f*^ϕ^ which represents the ionic currents across the cell membrane and the conductivity tensor *****D***** that accounts for a fast signal propagation of *D*^||^ parallel to the cardiac muscle fiber direction *****f***** and a slow signal propagation *D*^⊥^ perpendicular to it (Clerc, 1976; Plank et al., 2008; Goktepe and Kuhl, 2009),

(2) $$\begin{array}{l} D=D^{\left|\right|}f⊗f+D^{⊥}\left[I-f⊗f\right] \end{array}$$

In general, the ionic currents *f*^ϕ^ are functions of the transmembrane potential ϕ and a set of state variables *****q*****(ϕ) (Wong et al., 2013; Lee et al., 2016), *f*^ϕ^(ϕ, *****q*****(ϕ);*t*) where the state variables themselves are governed by ordinary differential equations, $\dot{q}=g\left(\phi ,q\left(\phi \right);t\right).$ The number of currents and state variables determines the complexity of the cell model and varies for different cell types. For human Purkinje fiber cells, we adopt the Stewart model (Stewart et al., 2009) which tracks 14 ionic currents using 20 state variables

(3) $$\begin{array}{l} \begin{array}{c} I_{\text{ion}}=I_{\text{CaL}}+I_{\text{Na}}+I_{\text{Cab}}+I_{\text{Nab}}+I_{\text{Kr}}+I_{\text{Ks}}+I_{\text{K}1}+I_{\text{to}}\\ \;+I_{\text{f}}+I_{\text{sus}}+I_{\text{NaK}}+I_{\text{pCa}}+I_{\text{pK}}+I_{\text{NaCa}} \end{array} \end{array}$$

To represent electrophyiological behavior of human ventricular cardiomyocytes, we adopt the O'Hara-Rudy model (O'Hara et al., 2011) with a minor modification (Priest et al., 2016) of the fast sodium current *I*_Na_ (ten Tusscher et al., 2004). Studies have shown that this *I*_Na_ substitution leads to a physiological conduction velocity restitution behavior, with a minor impact on the action potential behavior (Elshrif and Cherry, 2014). The resulting model tracks 15 ionic currents defined through a total of 39 state variables,

(4) $$\begin{array}{l} \text{​​​​​​}I_{\text{ion}}=I_{\text{CaL}}+I_{\text{Na}}+I_{\text{CaNa}}+I_{\text{CaK}}+I_{\text{Cab}}+I_{\text{Nab}}+I_{\text{Kb}}+I_{\text{Kr}}\\ \;+I_{\text{Ks}}+I_{\text{K}1}+I_{\text{to}}+I_{\text{NaK}}+I_{\text{pCa}}+I_{\text{NaCa},\text{i}}+I_{\text{NaCa},\text{ss}}\; \end{array}$$

To incorporate drug effects into our multiscale models, we selectively block the relevant ionic currents in the Purkinje and cardiomyocyte cell models (Sahli Costabal et al., 2018b). These blocks are informed by experimental patch-clamp experiments that study the fractional blockage β of different ion channels at varying drug concentrations (McMillan et al., 2017). We implement these fractional blockings using fitted Hill-type equations of the form,

(5) $$\begin{array}{l} \beta =\frac{C^{h}}{{IC}_{50}^{h}+C^{h}} \end{array}$$

which are characterized by the exponent *h* and the concentration ${IC}_{50}^{h}$ required to achieve a 50% current block. To apply a specific drug, we select a desired concentration *C*, calculated the fractional blockage β_ion_ for each considered ion channel, and scale the corresponding ion current channels by the fractional blockage [1 − β],

(6) $$\begin{array}{l} I_{\text{ion}}^{\text{drug}}=\left[1-\beta \right]I_{\text{ion}} \end{array}$$

### 2.2. Sex-Specific Cardiac Electrophysiology

#### 2.2.1. Sex-Specific Subcellular Ion Channel Activity

We deduced sex-based differences in ventricular ion channel activity from the expression level of key cardiac ion channel subunit proteins, quantified using western blotting, and genes, assessed through polymerase chain reaction analysis, in endo- and epicardial ventricular tissue from non-diseased explanted male and female human hearts (Gaborit et al., 2010). More specifically, we use the protein expression of Na_V_1.5 to scale the late sodium current *I*_NaL_ ion channel activities, the mRNA expression of ATP2B4 (Ca^2+^ ATPase 4) to scale the sarcolemmal calcium pump current *I*_pCa_, the protein expression of hERG to scale the rapid delayed rectifier potassium current *I*_Kr_, the protein expression of MinK to scale the slow delayed rectifier potassium current *I*_Ks_, the mRNA expression of KCNJ4 (Kir2.3) to scale the inward rectifier potassium current *I*_K1_, the mRNA expression of SLC8A1 (NCX1) to scale the sodium calcium exchange currents *I*_NaCa,i_ and *I*_NaCa,ss_, the mRNA expression of ATP1A1 and ATP1A3 (Na^+^/K^+^ ATPase α1 and α3) expression to scale the sodium potassium pump current *I*_NaK_, and the mRNA expression of KCNA5 (K_V_1.5) expression to scale the background potassium current *I*_Kb_. Moreover, we use the the mRNA expression of the RYR2 gene to scale the activity of the Ca^2+^ release channels, the mRNA expression of ATP2A2/SERCA2 (Ca^2+^ ATPase 2) to scale the activity of the Ca^2+^ uptake channels, and the mRNA expression of CALM3 expression to scale the Ca^2+^ buffering capacity through the calmodulin 3 concentration [CMDN]. Table A1 provides an in-depth overview of the sex-specific and transmurally varying mRNA/protein expression data. To deduce ion channel activities from the ion channel subunit expression, we followed transcriptional and functional scaling rules (O'Hara et al., 2011; Yang and Clancy, 2012).

The baseline endocardial O'Hara-Rudy model was developed, calibrated and thoroughly validated on experimental data collected from non-diseased ventricular tissue of 140 human donors, of which 78 were male. Therefore, we consider this baseline model to be a linear interpolated, 56% male and 44% female representation of the sex-specific representative endocardial cell models. By applying this linear interpolation rule to the aforementioned sex-specific mRNA and protein expression in the endocardial tissue, we computed the sex-specific ion channel activity ratio for the endocardial cardiomyocytes disclosed in Table 1. These ratios are relative scalings to the ion channel conductivities of the baseline endocardial model. Based on the transmural electrophysiological heterogeneity of the healthy human myocardial wall (Drouin et al., 1995; Glukhov et al., 2010; Okada et al., 2011), we parameterize three different transmural cell types: endocardial, midwall, and epicardial cells (O'Hara et al., 2011). To prescribe the epicardial ion channel activity, we use the reported relative epi/endo mRNA and protein expression data (Gaborit et al., 2010), following the expression/current activity correlations discussed before. To define the midwall ion channel activity, we implement relative mid/endo and epi/mid ratios (O'Hara et al., 2011). These ratios were deduced from reported epicardial vs. midwall protein expression data (Szabó et al., 2005) and midwall vs. endocardial mRNA expression data (Soltysinska et al., 2009). Finally, the midwall and epicardial activity of the transient outward potassium channel *I*_to_ was scaled based on functional patch-clamp data collected on myocytes isolated from the human non-failing left ventricle (Näbauer et al., 1996). The complete set of sex-specific and transmurally varying ion channel activity ratios relative to the baseline endocardial model can be found in Table 1. Given the current lack of an extensive experimental human dataset on genetic, transcription, or functional sex differences in ion channel activity for Purkinje fibers, we do not introduce any sex-specific ion channel scaling in the baseline Purkinje cell model by Stewart et al. (2009).

**Table 1:** Sex-specific subcellular ion channel activity.

	**Male**			**Female**		
	**Epi**	**Mid**	**Endo**	**Epi**	**Mid**	**Endo**
*I*_NaL_	0.77	1.06	1.06	0.65	0.93	0.93
*I*_to_	4.00	4.00	1.00	4.00	4.00	1.00
*I*_pCa_	0.70	1.97	0.79	1.26	3.17	1.27
*I*_Kr_	1.20	0.88	1.10	0.96	0.70	0.87
*I*_Ks_	1.16	1.10	1.10	0.93	0.88	0.88
*I*_K1_	1.07	1.39	1.07	0.76	1.18	0.91
*I*_NaCa,i_ / *I*_NaCa,ss_	1.10	1.41	1.01	1.07	1.38	0.99
*I*_NaK_	0.92	0.70	1.00	0.87	0.70	1.00
*I*_Kb_	0.82	1.25	1.25	0.42	0.68	0.68
Ca^2+^ release	1.13	1.68	0.99	0.89	1.72	1.01
Ca^2+^ uptake	1.33	0.94	0.94	1.85	1.08	1.08
[CMDN]	0.97	0.92	0.92	1.28	1.11	1.11

***Sex-specific subcellular ion channel activity**. Sex-specific and transmurally varying subcellular ion channel activity scaling used in computational models based on mRNA and protein ion channel subunit expression and functional data (Näbauer et al., 1996; Szabó et al., 2005; Soltysinska et al., 2009; Gaborit et al., 2010; O'Hara et al., 2011; Yang and Clancy, 2012)*.

The baseline Purkinje and sex-specific endo-, mid- and epicardial temporal transmembrane potential evolutions is computed by solving Equations (3) and (4), and their intrinsic systems of ordinary differential equations governing channel-specific gating variables in Myokit (Clerx et al., 2016). To achieve a steady state, we prepace each cell type for 1,000 cycles at a frequency of 1 Hz and then simulate five additional beats. To study the cellular restitution behavior, we compare the action potential duration at 90% repolarization after steady state S1 pacing at cycle length 1,000 ms, followed by a single S2 extrasystolic stimulus delivered at various diastolic intervals ranging between 0 and 1,000 ms.

#### 2.2.2. Sex-Specific Tissue Conductivity

We introduce tissue-level differences between both sexes by rescaling the average anisotropic conductivities parallel, *D*^||^, and perpendicular, *D*^⊥^, to the myofiber directions *****f*****. These scalings are informed by the sex-specific mRNA expression of connexin43, the primary ventricular gap-junction subunit (Dhillon et al., 2013). Assuming *D*^||^ = 0.090 mm^2^/ms and *D*^⊥^ = 0.012 mm^2^/ms (Niederer et al., 2011) represents the anisotropic conductivity in the average, 50% male / 50% female, human heart, the 50% higher connexin43 expression in male vs. female cardiomyocytes (Gaborit et al., 2010) leads to *D*^||^ = 0.108 mm^2^/ms and *D*^⊥^ = 0.014 mm^2^/ms, and *D*^||^ = 0.072 mm^2^/ms and *D*^⊥^ = 0.010 mm^2^/ms, for male and female myocardial tissue, respectively.

#### 2.2.3. Sex-Specific Organ Geometry

**Figure 2:**
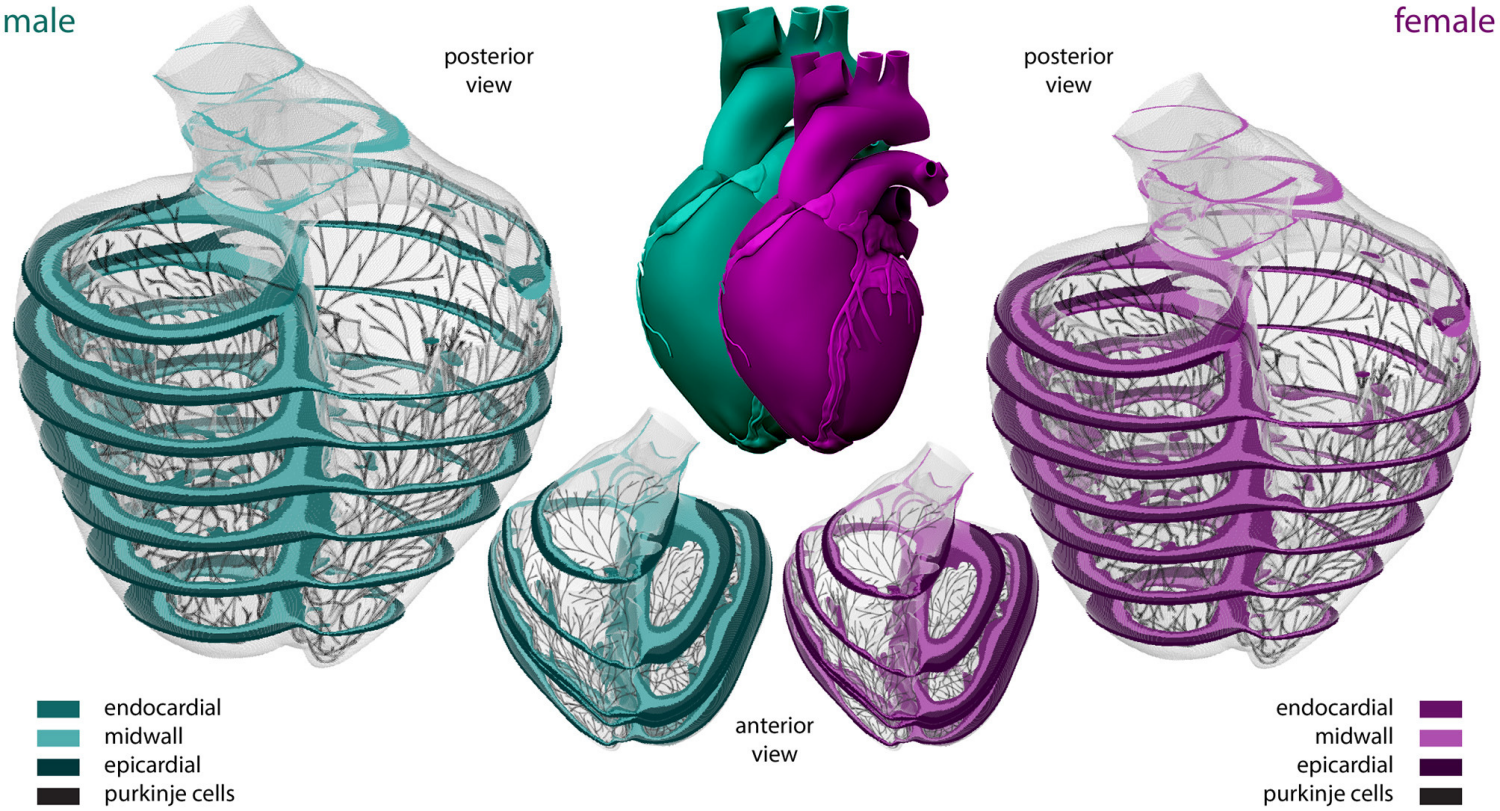
**Sex-specific multiscale exposure-response simulators**. Male and female human heart model created from high resolution magnetic resonance images of a healthy male adult and isogeometrically scaled according to the average adult male/female ventricular mass ratio. The ventricular walls are discretized with 6,878,459 regular linear hexagonal finite elements. The Purkinje fiber networks are discretized with 39,772 linear cable elements and are connected to the ventricles at their terminals through 3,545 resistor elements. Endocardial, midwall, and epicardial cells are marked in median, light and dark green and purple, respectively; Purkinje cells are shown in black. Long-axis transmural slices are shown in the anterior view representations. Short-axis transmural slices are shown in the enlarged posterior views.

To model the multiscale cardiac electrophysiological behavior across the male and female heart, respectively, we discretize the governing Equations (1)–(4) in space using finite elements (Goktepe and Kuhl, 2009) and in time using finite differences (Sahli Costabal et al., 2018a). Temporally, we utilize an explicit time integration scheme for both the reaction-diffusion equation (Equation 1) and the Purkinje and cardiomyocyte (Equations 3 and 4) ionic models, with a fixed time step size Δ*t*= 0.005 ms. Spatially, we use a full three-dimensional representation of the human ventricles, created from magnetic resonance images of a healthy, 21-year old, 50th percentile U.S. male (Baillargeon et al., 2014; Zygote Media Group Inc., 2014; Peirlinck et al., 2021). We infer the female geometry as a 90% isometric scaling of the male geometry, following the reported average female to male adult left ventricular mass ratio of 72% (de Simone et al., 1995). Both geometries are subdivided using linear hexagonal finite elements with a constant edge length of 0.3 and 0.27 mm for the male and female model, respectively. This results in 6,878,459 regular linear hexagonal finite elements, with a total of 7,519,918 nodes. By solving a series of Laplace problems with different essential boundary conditions on this solid mesh (Perotti et al., 2015), we incorporate the transmural heterogeneity of the ventricular wall as showcased in Figure 2. This 20% endocardial, 30% midwall, 50% epicardial tissue arrangement ensures positive T-waves to simulate a healthy baseline electrocardiogram (Okada et al., 2011). In a similar fashion, we assign local myofiber orientations *****f***** to each and every element, accounting for the heart's intrinsic myofiber architecture (Lombaert et al., 2012; Peirlinck et al., 2018). We generate the Purkinje fiber network by growing a fractal tree on the endocardial surface of the heart (Sahli Costabal et al., 2015), and discretize it using 39,772 linear cable elements and 39,842 nodes. The terminals of this network are connected to the ventricular myocardium using 3545 resistor elements with a resistance of 1.78Ω*m* (Niederer et al., 2011). We excite the Purkinje network at the atrioventricular node every second, and study the excitation profile of the heart over a period of 5,000 ms. To solve the resulting system of equations, we use the finite element software package Abaqus (Dassault Systèmes Simulia Corp., 2020), typically taking 24 h using 240 CPUs (Towns et al., 2014). In this verified implementation (Niederer et al., 2011; Sahli Costabal et al., 2019c), we exploit the structural similarities between the continuum equations and a classical heat transfer problem with a non-linear heat source (Sahli Costabal et al., 2018a).

Finally, we post-process the spatiotemporal excitation profiles to calculate pseudo-electrocardiograms ϕ_e_(*****x*****_e_) at a virtual precordial electrode location *****x*****_e_ two centimeters away from the left ventricular wall (Kotikanyadanam et al., 2010; Sahli Costabal et al., 2018b). In summary, at every point *****x***** of the heart, we project the gradient of the transmembrane potential ∇ϕ onto the direction vector ∇(1/||*****r*****||) with *****r***** = *****x*****_e_ − *****x*****, and integrate this projection across the entire cardiac domain Ω: $\phi_{\text{e}}\left(x_{\text{e}}\right)=-\int_{\Omega }\nabla \phi \cdot \nabla \left(1/||r||\right)\text{dV}$. We manually labeled the electrophysiological behavior as non-arrhythmogenic or arrhythmogenic, based on the absence or presence of non-regular chaotic twisting QRS complexes during the last five simulated beats. These electrocardiographic hallmarks of arrhythmogenesis are caused by regional early afterdepolarizations overtaking the regular depolarization wave initiated by the Purkinje network.

### 2.3. Data-Driven Arrhythmogenic Risk Classification

Using the male and female multiscale cardiac electrophysiology models, we develop two sex-specific arrhythmogenic risk classifiers based on drug- and dose-specific ion channel blockage. Given the high computational cost of evaluating arrhythmogenesis for a single full organ-scale and drug-induced ion-channel blockage combination, we combine multiple machine learning techniques to create and train sex-specific arrhythmogenic drug risk classifiers. We first narrow the drug effect parameter space by studying the cellular drug sensitivity to various ion channel blockings. For this sensitivity analysis, we use multivariable logistic regression techniques, as they have been proven to be computationally more efficient than one-at-a-time parameter sensitivity analyses (Lee et al., 2013) and highly suitable for studying processes with binary outcomes (Morotti and Grandi, 2017). Next, we apply the developed sex-specific high-fidelity multiscale exposure-response simulators to quantify the risk of drug-induced arrhythmogenesis within the identified critical drug-induced ion channel blocking parameter space. To reduce the computational cost of exploring this parameter space, we develop and train multi-fidelity risk classifiers that have been shown to outperform single high-fidelity risk classifiers (Sahli Costabal et al., 2019b). More specifically, we combine low-fidelity mid-wall cardiomyocyte simulations and high-fidelity heart simulations to train a Gaussian process classifier that characterizes the probability of arrhythmogenicity based on the two most important ion channel blockage features for arrhythmogenesis. Using active learning, we maximize the information gained by each possible low-and high-fidelity sample we evaluate, keeping the computational costs of training our arrhythmogenic risk classifiers as low as possible.

#### 2.3.1. Sensitivity to Drug-Induced Ion Channel Blockage

To explore the male and female arrhythmogenic sensitivity to drug-induced ion channel blocking in a computationally tractable way, we focus on seven specific ion channel currents *I*_Kr_, *I*_Na_, *I*_NaL_, *I*_CaL_, *I*_Ks_, *I*_to_, and *I*_K1_ identified to be important in both depolarization and repolarization of the cardiac action potential (Crumb et al., 2016; Fermini et al., 2016). As it has been shown that early afterdepolarizations and repolarization abnormalities are a precursor of arrhythmia at the cellular level (Qu et al., 2013), we identify which channels have the most significant impact on de- and repolarization abnormality development (Sahli Costabal et al., 2020). We systematically create 10,000 cellular drug-blocking samples by performing Latin hypercube sampling on a seven-dimensional blockage parameter space [0.0, 0.95]^7^, resulting in a sample set $B=\left\{\beta_{\text{Kr}},\beta_{\text{Na}},\beta_{\text{NaL}},\beta_{\text{CaL}},\beta_{\text{Ks}},\beta_{\text{to}},\beta_{\text{K1}}\right\}\in {\left[0.0,0.95\right]}^{10,000\times 7}$. For each sample, we pre-pace the male and female cell model for 1,000 cycles at a frequency of 1Hz, and subsequently simulate the corresponding ion channel blockage effect on the cardiomyocyte action potential. We do this for the male and female midwall cells, as previous work has shown that arrhythmogenic risk assessment is particularly sensitive to midwall cell distributions (Antzelevitch and Sicouri, 1994; Sahli Costabal et al., 2018b). For each sample, we define de- or repolarization abnormalities as the occurrence of a change in potential greater than 0.1 mV/ms, or the transmembrane potential not dropping below −40 mV, between the 50 and 1,000 ms time marks of each beat (Sahli Costabal et al., 2019c). Subsequently, we perform a male and female logistic regression trained on the blockage samples and the post-processed absence/presence of abnormalities. By extracting the marginal effects (Norton et al., 2019), we quantify the arrhythmogenic risk of each channel blockade and select the two most important opposing anti-arrhythmic and pro-arrhythmic ion channel blocking feature β_−_ and β_+_ for arrhythmogenic risk classification.

#### 2.3.2. Gaussian Process Risk Classification

##### Single-Fidelity Gaussian Process Classifier

We use physics-based electrophysiological modeling (section 2.1) to generate a dataset comprised of cell- or whole heart input/output pairs

(7) $$\begin{array}{l} D=\left\{{\left(x_{i},y_{i}\right)}_{i=1}^{N}\right\}=\left\{X,y\right\}. \end{array}$$

Here, the inputs *****x*****_*i*_ contain the two most important drug-induced ion channel blocking arrhythmogenicity features brought forward in section 2.3.1. We set the most anti-arrhythmic β_−_ and pro-arrhythmic β_+_ ion channel blocking feature to vary between 0 and 95%. As such, $X=\left\{\beta_{-},\beta_{+}\right\}\in {\left[0.0,0.95\right]}^{N\times 2}$ for *N* training samples. In this arrhythmogenic risk assessment, the outputs *y*_*i*_ can only take on two binary values: zero and one, representing the absence or presence of de- and repolarization abnormalities for cell level simulations and arrhythmogenesis for whole heart simulations. As such, *****y***** ∈ {0, 1}^*N*^.

To set up the Gaussian process classifier, we put forward a latent function *f*(*****x*****) (Rasmussen, 2004) and standardize our dataset D so we can work with a zero-mean Gaussian process (GP) prior of the form

(8) $$\begin{array}{l} f\sim \mathrm{GP}\left(0,k\left(x,x^{\prime };\theta \right)\right). \end{array}$$

Here, *k*(·, ·;***θ***) is a covariance kernel function depending on a set of parameters ***θ***, which we will determine using Bayesian inference, ex infra. By passing the Gaussian process output *f* through a logistic sigmoid warping function σ, we constrain the output to [0, 1]. These outputs entail meaningful class probabilities.

To set up our Bayesian inference framework, we define the conditional class probability as

(9) $$\begin{array}{l} \pi \left(x\right)=p\left(y=1|x\right)=\sigma \left(f\left(x\right)\right) \end{array}$$

and assume the class labels are independent according to a Bernoulli likelihood with probability σ(*y*) (Nickisch and Rasmussen, 2008). Following our prior work (Sahli Costabal et al., 2019b), we choose an automatic-relevance determination squared exponential kernel,

(10) $$\begin{array}{l} k\left(x,x^{\prime };\theta \right)=\eta \exp\left[-\overset{M}{\underset{m=1}{\sum }}{\left(x_{m}-x_{m}^{\prime }\right)}^{2}/\left(2\ell_{m}^{2}\right)\right] \end{array}$$

parameterized by ***θ***: = {η, ℓ_1_, …, ℓ_*M*_}. We set η ~ HalfNormal(σ = 5) and ℓ_*m*_ ~ Gamma(α = 2, β = 2) for *m* = 1, …, *M* length scales as weakly informative prior distributions. Lacking an analytic solution for the posterior distribution, we resort to approximate-inference techniques to calibrate this model on the available generated data. Here, we use the NO-U-Turn sampling algorithm (Hoffman and Gelman, 2014), which is a self-tuning Markov Chain variant of Hamiltonian Monte Carlo, as implemented in PyMC3 (Salvatier et al., 2016).

To utilize the Gaussian process classifier for arrhythmogenic risk stratification, we use the resulting posterior ***θ*** distribution to make class predictions y^*^ at new locations x^*^. We first compute the predictive random variable f^*^(x^*^) using the covariance matrix *****K***** ∈ ℝ^*N*×*N*^, which we obtain from evaluating the kernel function at the location of the input training data. Next, we sample f^*^ from the estimated posterior distributions. Finally, we run these f^*^ evaluations through the logistic sigmoid function σ to obtain a distribution of class probabilities y^*^ (Sahli Costabal et al., 2019b).

##### Multi-Fidelity Gaussian Process Classifier

We employ physics-based electrophysiological modeling (section 2.1) to generate a dataset

(11) $$\begin{array}{l} \begin{array}{c} D=\left\{\left[{\left(x_{L_{i}},y_{L_{i}}\right)}_{i=1}^{N_{L}}\right],\left[{\left(x_{H_{i}},y_{H_{i}}\right)}_{i=1}^{N_{H}}\right]\right\}\\ \;=\left\{\left[X_{L},X_{H}\right],\left[y_{L},y_{H}\right]\right\}=\left\{X,y\right\} \end{array} \end{array}$$

comprised of *N*_*L*_ low-fidelity midwall cell input/output pairs and *N*_*H*_ high-fidelity whole heart input/output pairs. Both low- and high-fidelity input sets explore the two-dimensional [0.0, 0.95]^2^ ion channel blockage parameter space identified in section 2.3.1. Both low-and high-fidelity outputs comprise binary variables *y*_*L*_*i*__, *y*_*H*_*i*__ = {0, 1}.

We model the cross-correlation structure between the low- and high-fidelity level using an autoregressive model for the latent function *f*_*H*_ (Kennedy, 2000),

(12) $$\begin{array}{l} f_{H}\left(x\right)=\rho f_{L}\left(x\right)+\delta \left(x\right) \end{array}$$

where ρ is a scalar parameter that needs to be inferred, capturing linear correlations between the high- and low-fidelity levels. The function δ aims to capture the bias in the predictions of the low-fidelity level. To complete the Gaussian model framework, we assume independent Gaussian priors for

(13) $$\begin{array}{l} \delta \sim \mathrm{GP}\left(0,k\left(x,x^{\prime };\theta_{H}\right)\right) \end{array}$$

(14) $$\begin{array}{l} f_{L}\sim \mathrm{GP}\left(0,k\left(x,x^{\prime };\theta_{L}\right)\right) \end{array}$$

where *k*(·, ·;***θ***_*H*_) and *k*(·, ·;***θ***_*L*_) are automatic-relevance determination squared exponential kernels conform Equation (10), resulting in parameters ***θ***_*H*_: = (η_*H*_, ℓ_*H*_1__, …, ℓ_*H*_*M*__), and ***θ***_*L*_: = (η_*L*_, ℓ_*L*_1__, …, ℓ_*L*_*M*__). To infer these parameters and the aforementioned scalar factor ρ, we set η_*H*_, η_*L*_ ~ HalfNormal (σ = 5), ℓ_*H*_*m*__, ℓ_*L*_*m*__ ~ Gamma(α = 2, β = 2) with *m* = 1, …, *M* length scales and ρ ~ Normal(μ = 0, σ = 10) as weakly informative prior distributions. We perform Bayesian inference following the same approach as for the single-fidelity Gaussian process classifier before.

#### 2.3.3. Active Learning

Given the high computational cost of our multiscale cardiac electrophysiology simulations, we apply an active learning strategy to maximally enhance the accuracy of our single- and multi-fidelity arrhythmogenic risk classifiers with a minimal amount of additional sample evaluations in the studied parameter space. More specifically, we exploit the posterior uncertainty estimates of our Bayesian models to select the next sampling point expected to increase the accuracy of our classifier the most. We pick the next sampling point based on the following minimization problem:

(15) $$\begin{array}{l} x^{\text{new}}=\underset{x\in X_{\text{cand}}}{\arg\min}\frac{\left|\hat{\mu }\left(x\right)\right|}{\sqrt{\hat{\Sigma }\left(x\right)}} \end{array}$$

where $\hat{\mu }$ and $\hat{\Sigma }$ are the Monte Carlo estimates of the mean and variance of *f*(*****x*****). Here, we apply Latin hypercube sampling to generate a set of 1,000 candidate locations *****X*****_cand_ to sample. Next, we compute the electrophysiological response and class label *y*^new^ for the selected sample *****x*****^new^, and add this input/output pair to the dataset. We subsequently re-train the classifier for this new dataset and repeat this process until we reach a well-defined classification border or computational resources are depleted (Sahli Costabal et al., 2019b).

#### 2.3.4. Multi-, Low-, and High-Fidelity Arrhythmogenic Risk Classification

We start by training a male and female single-fidelity classifier based on low-fidelity mid-wall cell simulations. We explore the input space with 25 Latin hypercube samples and evaluate whether or not the resulting ion channel blockings lead to de- or repolarization abnormalities as defined in section 2.2.1. We train a single-fidelity classifier based on this dataset D (Equation 7) and further explore and exploit the resulting low-fidelity arrhythmogenesis classification boundaries using 25 additional active learning samples.

Next, we combine the 50 low-fidelity input/output pairs with 10 Latin hypercube drug blocking sample evaluations of the full heart models as described in section 2.2.3. We use this combined low- and high-fidelity dataset D (Equation 11) to train a multi-fidelity arrhythmogenic risk classifier. Subsequently, we improve the accuracy of the classification boundary using 15 additional high-fidelity active learning sample evaluations.

### 2.4. Drug Risk Stratification

Using our multi-fidelity arrhythmogenicity classification boundary, we estimate the arrhythmogenic risk of three drugs, a high, intermediate and low risk drug (Li et al., 2018), by computing the critical drug concentration at which arrhythmia will start developing. We select three drugs for which the concentration-block response curve is well-described (McMillan et al., 2017) for the two cardiac currents that have the most significant impact on arrhythmogenic risk prediction (section 2.3.1). The critical drug concentration is found at the intersection of the multi-fidelity arrhythmogenesis classification boundary and the two-dimensional concentration-block trajectory described by Equation (5). If the drug's concentration-block trajectory does not cross the risk boundary, that drug can be considered safe for the studied sex.

## 3. Results

### 3.1. Sex-Specific Cardiac Electrophysiology

#### 3.1.1. Cell Level Differences

**Figure 3:**
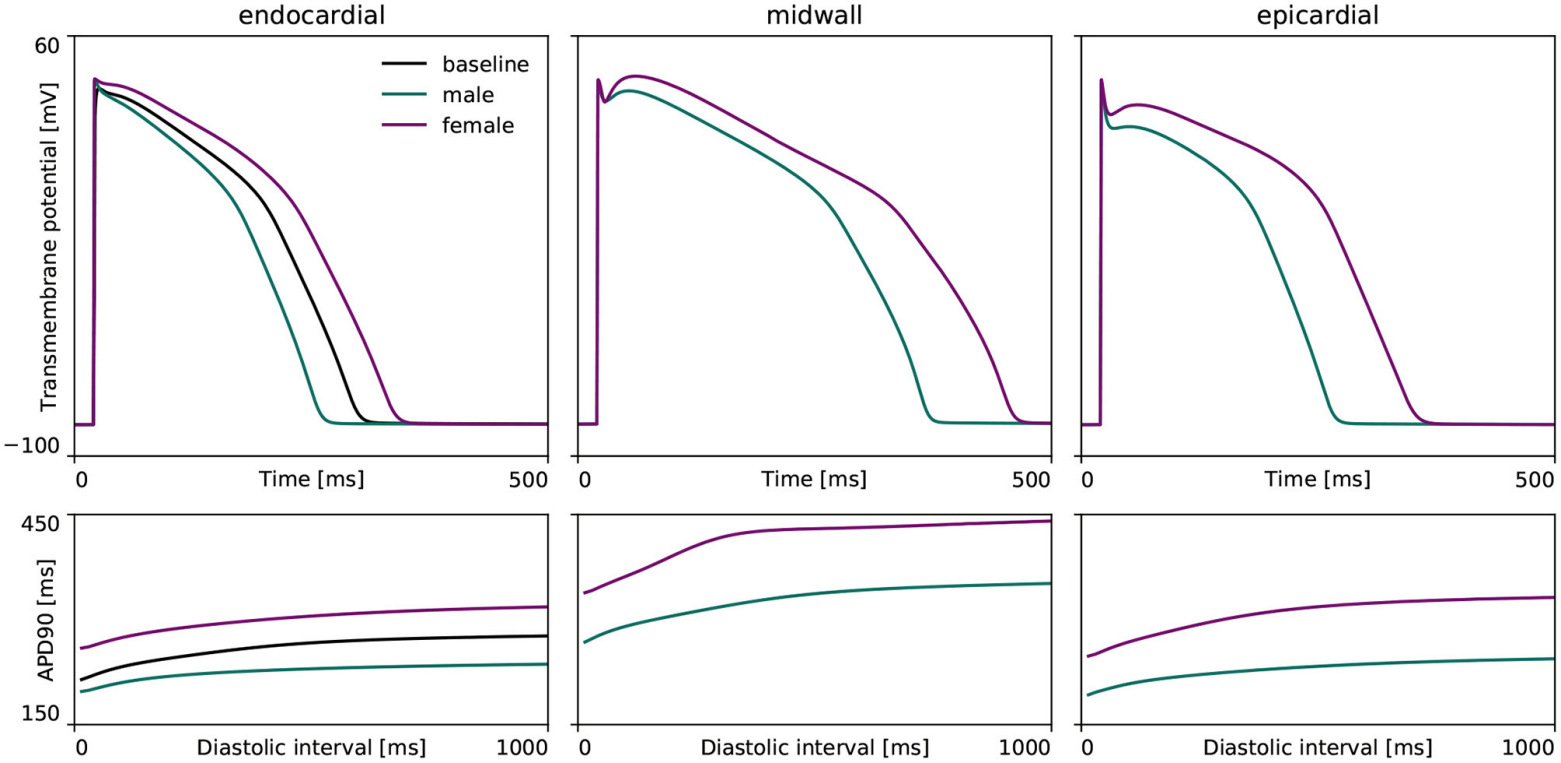
**Sex differences in transmural ventricular cardiomyocyte behavior**. Sex-specific differences in endocardial, midwall and epicardial ventricular action potentials based on the experimentally quantified differences in ion channel activity. The green and purple lines represent the male and female steady state action potentials **(Top)** and action potential duration restitution curves **(Bottom)** for each transmural cell line. For the endocardial cell, the baseline steady-state action potential evolution and restitution curve of the calibrated and validated O'Hara-Rudy model for the 56% male / 44% female mixed-population is shown in black.

Figure 3 highlights the sex differences in electrophysiological behavior for endocardial, midwall and epicardial cells based on the experimentally quantified ion channel activity discussed in section 2.2.1. Here, the green and purple lines represent the male and female action potential evolutions, respectively. The black line in the endocardial cell subplot represents the action potential profile for the baseline O'Hara-Rudy model for the endocardial cell, which results from the underlying 56/44% interpolation of the male and female ion channel activities disclosed in Table 1. Relative to male cells, the female sex-specific baseline action potential durations are substantially larger for all transmural cell types. More specifically, the male and female endocardial action potential duration at 90% repolarization at 1 Hz pacing amounts to 233 and 314 ms, respectively. Similarly, it takes 309 and 379 ms for male and female midwall cells, and 221 and 296 ms for male and female epicardial cells to repolarize, respectively. The male and female endocardial action potential duration restitution amounted to 208 and 270 ms at a diastolic interval of 100 ms, to 217 and 284 ms at a diastolic interval of 200 ms, and 229 and 309 ms at a diastolic interval of 500 ms. Similarly, male and female action potential duration restitution for the midwall cell lines amounted to 262 and 348 ms, 275 and 358 ms, and 301 and 371 ms for diastolic intervals 100, 200, and 500 ms, respectively. For the epicardial cell lines, we computed male and female action potential duration restitutions of 204 and 272 ms, 215 and 280 ms, and 233 and 288 ms for diastolic intervals 100, 200, and 500 ms, respectively.

#### 3.1.2. Organ Level Differences

**Figure 4:**
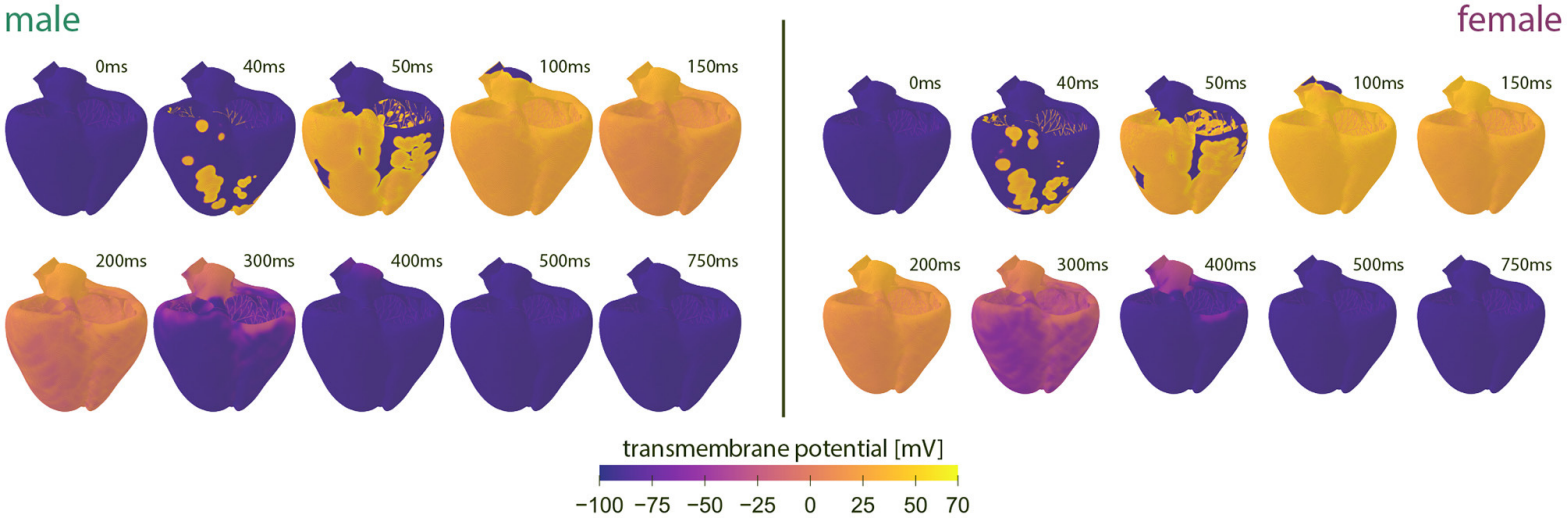
**Baseline spatiotemporal excitation profiles for the male and female heart**. Evolution of the transmembrane potential for the male and female heart without drugs. Snapshots are taken from the last simulated beat. During depolarization, the Purkinje fibers drive the sharp depolarization front from apex to base. During repolarization, both ventricles gradually return to their resting state. The combined sex-differences in ion channel activity, tissue conductivity and organ-scale geometry lead to a slower depolarization in the female heart than the male heart.

**Figure 5:**
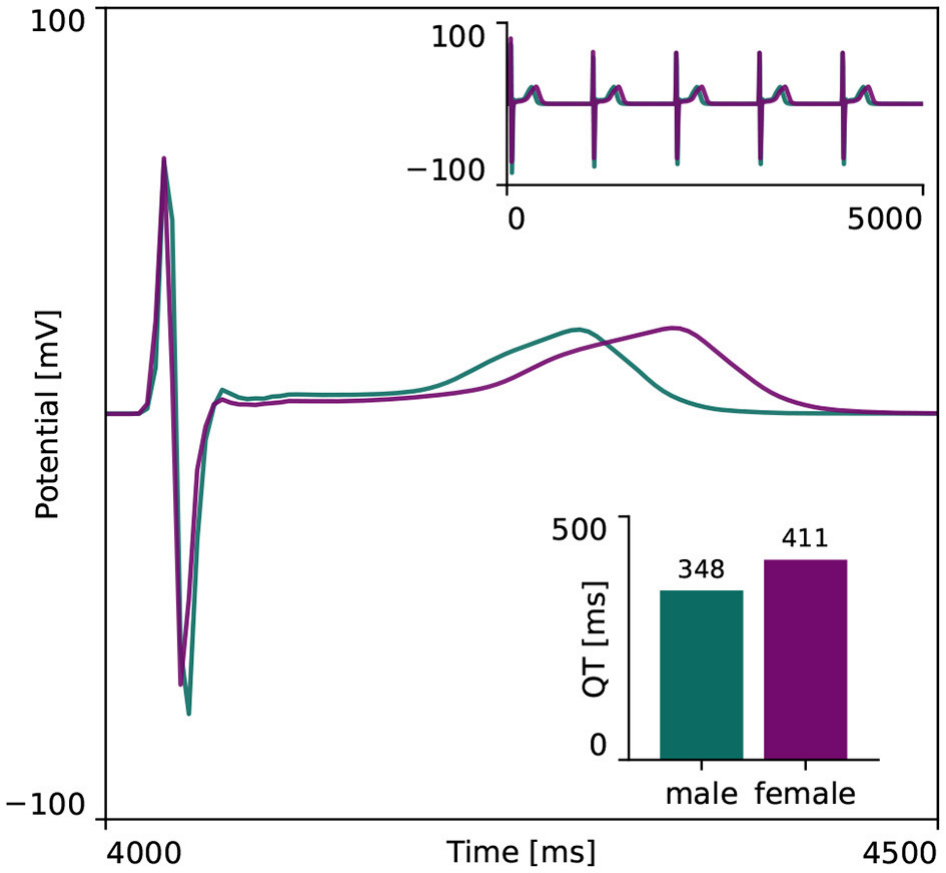
**Baseline electrocardiogram recordings for the male and female heart**. Electrocardiogram recordings for the male and female heart models without drugs. Both male and female electocardiograms display regular periodic activation patterns, as shown in the upper right inlay plot. The repolarization delay between the male and female heart is shown in more detail in the main plot, focusing on the first 500 ms of the last simulated beat. The resulting male and female QT interval amounted to 348 and 411 ms, respectively, as shown in the lower right inlay plot.

Figure 4 showcases the baseline spatiotemporal excitation profile for the male and female heart. The ten snapshots illustrate the combined effect that sex-differences in subcellular ion channel activity, tissue-level conductivity and organ-scale geometry have on the spatiotemporal transmembrane potential evolution, without the effect of any drugs. In both the male and female heart, the Purkinje network drives a sharp depolarization front propagating rapidly from apex to base and across the heart. At 100 ms, both the male and female ventricles are fully excited. In the male heart, the repolarization phase, during which the heart returns to its resting state, is finished between 300 and 400 ms. For the female heart, this repolarization takes longer, finishing between 400 and 500 ms. The exact duration between the beginning of the depolarization and the end of the repolarization is showcased in the electrocardiogram recordings computed for the male and female baseline heart model in Figure 5. The QRS complex lasts 73 and 69 ms for the male and female heart, respectively. This difference in QRS duration was mainly driven by the smaller female vs. male heart size and reduced conductivity. In parallel, the prolongation of the T wave with respect to the end of the QRS complex results mainly from the sex-specific differences in ion channel activity at the subcellular level. The multiscale combined effect of these sex differences amounts to QT intervals of 348 and 411 ms for the male and female baseline heart, respectively.

### 3.2. Sex-Specific Drug-Response Characteristics

**Figure 6:**
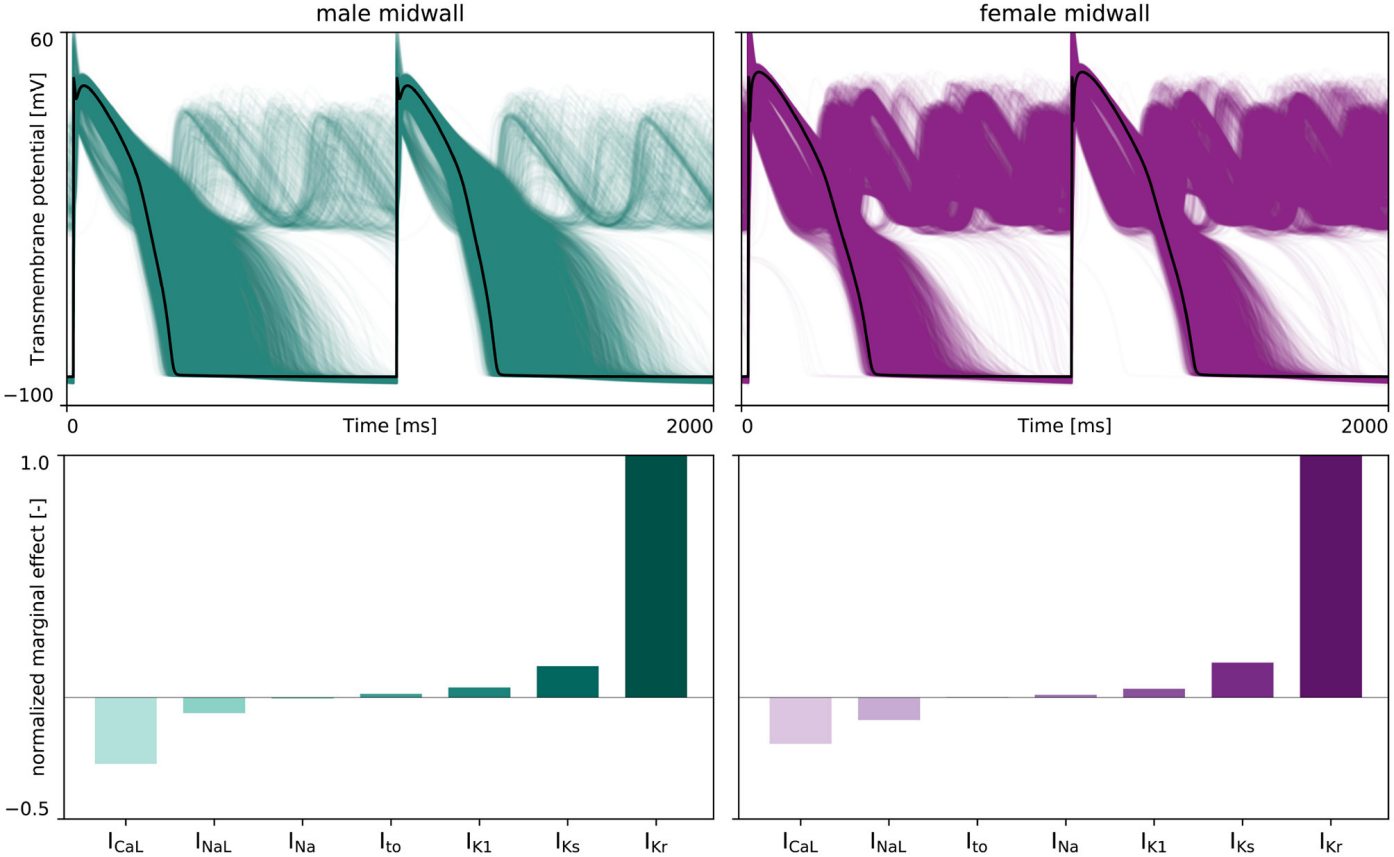
**Sex-specific sensitivity analysis drug-induced ion channel blocking on de- and repolarization abnormalities**. **(Upper)** The effect of drug-induced ion channel blocking on the male **(left)** and female **(right)** midwall transmembrane potential evolution. The black lines represent the baseline male and female action potential, without any ion channel blocking. The green and purple lines represent the transmembrane potential evolution for 5,000 distinct *I*_Kr_, *I*_Na_, *I*_NaL_, *I*_CaL_, *I*_Ks_, *I*_to_, and *I*_K1_ ion channel blocking combinations. **(Lower)** Normalized marginal effects of ion channel blocking on early afterdepolarizations development in male **(left)** and female **(right)** midwall cells. Negative normalized marginal effects highlight ion channel blocking leading to anti-arrhythmic effects, whilst positive marginal effects highlight the ion channels for which drug-induced blocking can have important pro-arrhythmic consequences.

Figure 6 represents the male and female anti- or pro-arrhythmic sensitivity to drug-induced ion channel blocking. As can be seen in the upper two plots, the female midwall cells are more sensitive to drug-induced ion channel blocking than male midwall cells. For the same set of ion channel blocking samples $B=\left\{\beta_{\text{Kr}},\beta_{\text{Na}},\beta_{\text{NaL}},\beta_{\text{CaL}},\beta_{\text{Ks}},\beta_{\text{to}},\beta_{\text{K1}}\right\}\in {\left[0.0,0.95\right]}^{10,000\times 7}$, we recorded 760 and 4,450 abnormalities for the male and female midwall cell, respectively.

The lower panel plots in Figure 6 depict the normalized marginal effects of drug-induced ion channel blocking on de- and repolarization abnormalities. For both male and female midwall cardiomyocytes, *I*_Kr_ blocking has a strong pro-arrhythmic effect, whilst *I*_CaL_ blocking has the largest anti-arrhythmic strength. The male and female normalized marginal effect of L-type *Ca*^2+^ channel blocking amounted to −0.272 and −0.190, respectively. As such, we selected drug-induced blocking of the rapid delayed rectifier potassium current, β_Kr_, and the L-type *Ca*^2+^ current channel, β_CaL_ as the two main pro-arrhythmic and anti-arrhythmic input features β_+_ and β_−_ for our male and female arrhythmogenic risk classifier.

### 3.3. Sex-Specific Risk Classifiers

**Figure A1:**
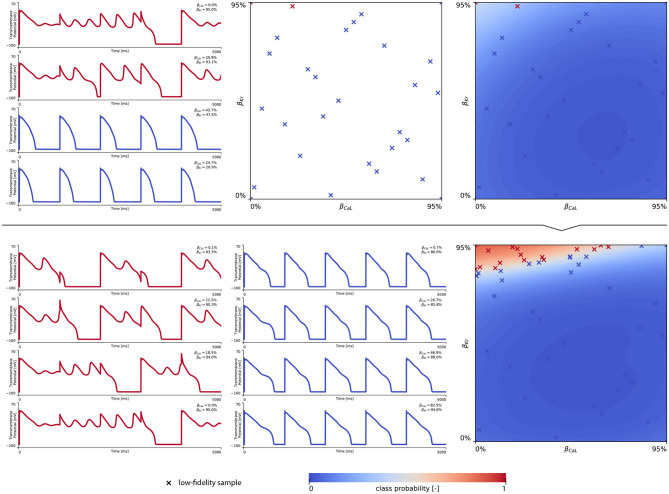
Male single-fidelity drug-induced risk classifier. **(Upper)** Initial exploration of the drug-induced de- and repolarization abnormality risk parameter space. The first 25 internal latin hypercube samples of the $B=\left\{\beta_{\text{Kr}},\beta_{\text{CaL}}\right\}$ parameter space were evaluated for male midwall cell abnormalities, as showcased in the upper middle plot. Transmembrane potential evolution profiles of two normal and two early depolarizing samples are shown on the upper left plot. Using this exploratory B dataset, an initial single-fidelity drug-induced risk classifier was trained for the male midwall cell, see upper right subplot. **(Lower)** Exploration and exploitation of the drug-induced risk classification boundary using active learning. The Gaussian process classifier was trained further using 25 additional active learning {β_Kr_, β_CaL_} samples. On the lower left, four active learning sample evaluations resulting in abnormalities are shown. The lower middle plot showcases four active learning sample evaluations that showed normal action potentials. The final male cellular drug-induced risk classification boundary is shown in the lower right plot.

**Figure A2:**
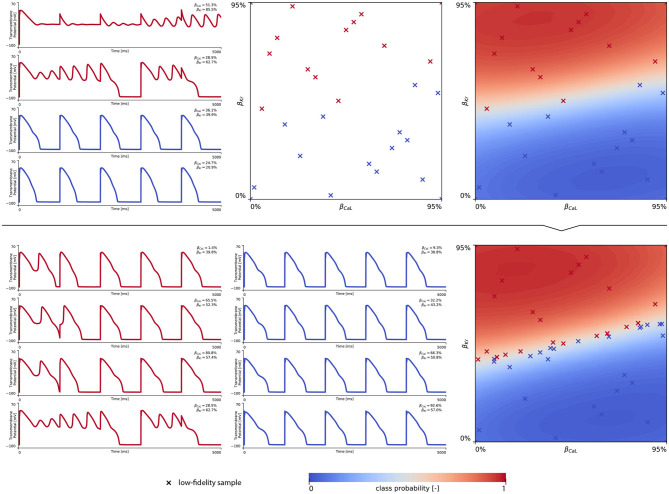
Female single-fidelity drug-induced risk classifier. **(Upper)** Initial exploration of the drug-induced de- and repolarization abnormality risk parameter space. The first 25 internal latin hypercube samples of the $B=\left\{\beta_{\text{Kr}},\beta_{\text{CaL}}\right\}$ parameter space were evaluated for female midwall cell abnormalities, as showcased in the upper middle plot. Transmembrane potential evolution profiles of two normal and two early depolarizing samples are shown on the upper left plot. Using this exploratory B dataset, an initial single-fidelity drug-induced risk classifier was trained for the female midwall cell, see upper right subplot. **(Lower)** Exploration and exploitation of the drug-induced risk classification boundary using active learning. The Gaussian process classifier was trained further using 25 additional active learning {β_Kr_, β_CaL_} samples. On the lower left, four active learning sample evaluations resulting in abnormalities are shown. The lower middle plot showcases four active learning sample evaluations that showed normal action potentials. The final female cellular drug-induced risk classification boundary is shown in the lower right plot.

In training our multi-fidelity arrhythmogenic risk classifiers, we first trained a single fidelity de- and repolarization classifier for the male and female midwall cell, respectively. Figures A1, A2 summarize the de- and repolarization abnormality classification boundary delineation in the studied {β_CaL_, β_Kr_} parameter space. The upper panel plots showcase the initial exploration phase to train these Gaussian process classifiers. The lower panel plots depict the subsequent exploration and exploitation phase through active learning.

**Figure 7:**
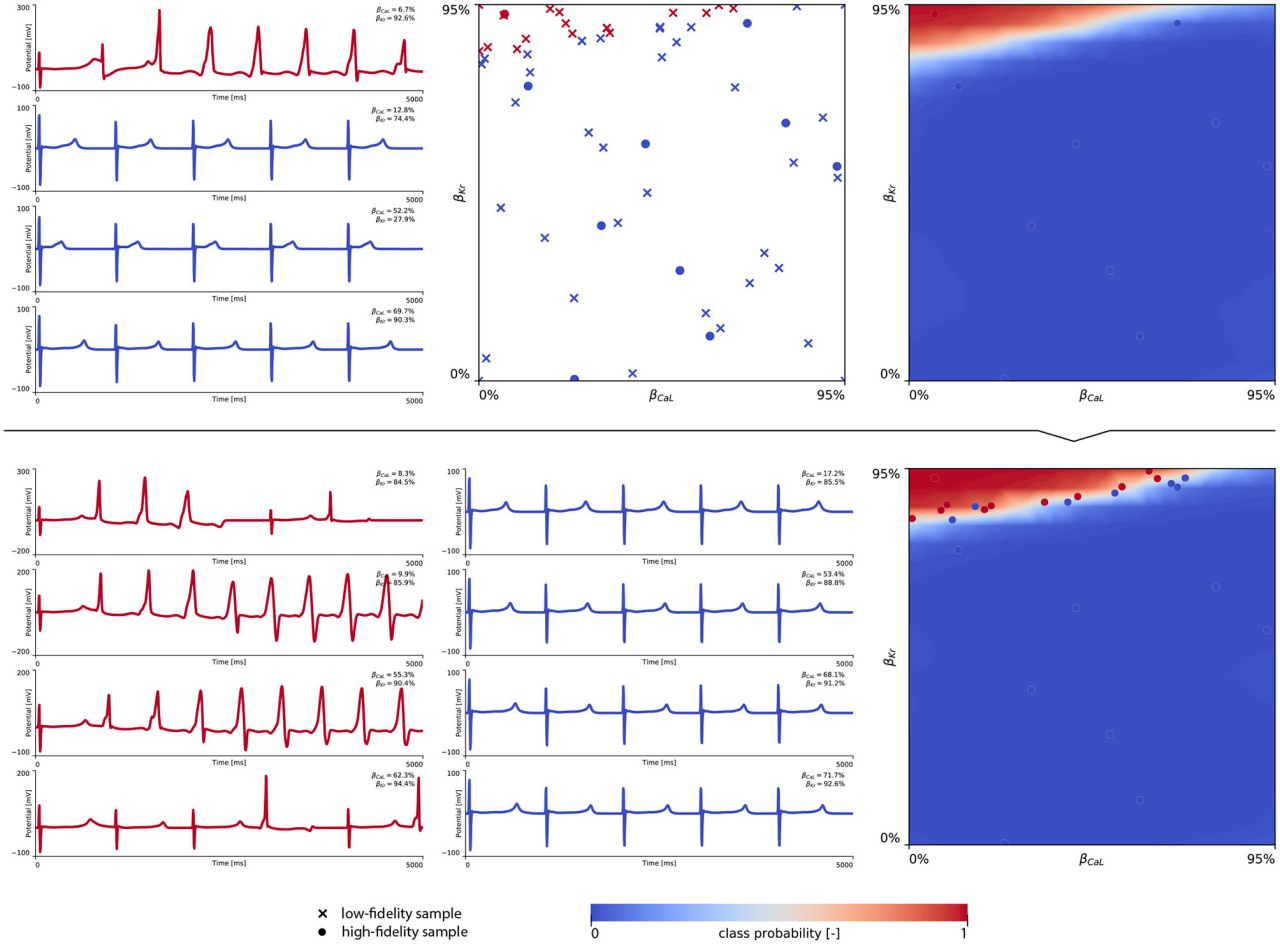
**Male multi-fidelity drug-induced arrhythmogenic risk classifier**. **(Upper)** Initial exploration of the male drug-induced arrhythmogenic risk parameter space. The first 10 internal Latin hypercube samples of the $B=\left\{\beta_{\text{Kr}},\beta_{\text{CaL}}\right\}$ parameter space were evaluated for male drug-induced arrhythmogenicity, as showcased in the upper middle plot with “dot” markers. Here, the low-fidelity samples from training the low-fidelity male midwall Gaussian process classifier are shown with “x” markers. Virtual electrocardiograms of one arrhythmic and three normal {β_Kr_, β_CaL_} samples are shown in the upper left column plots. The resulting drug-induced arrhythmogenicity probabilities for the male heart is shown in the upper right plot. **(Lower)** Active learning exploration and exploitation of the male drug-induced arrhythmogenic risk classification boundary. The multi-fidelity Gaussian process classifier was trained further using 15 additional active learning high-evaluations of {β_Kr_, β_CaL_} effects. On the lower left, four additional arrhythmic sample evaluations are shown. The lower panel middle plot showcases four additional sample evaluations showcasing normal heartbeats. The final male multi-fidelity drug-induced arrhythmogenicity classification boundary is shown in the lower right plot.

**Figure A3:**
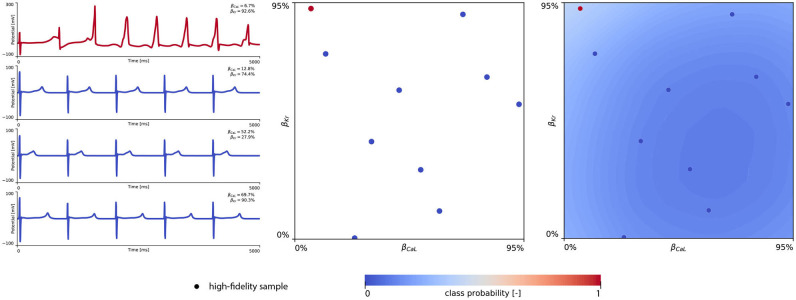
Male single-fidelity multiscale arrhythmia risk classifier. Initial single high-fidelity exploration of the male drug-induced arrhythmogenic risk parameter space. The middle plot showcases 10 internal latin hypercube {β_Kr_, β_CaL_} sample evaluations of arrhythmogenesis in the male heart. The virtual electrocardiograms for one arrhythmogenic and three non-arrhythmic sample evaluations are shown in the left plots. The resulting exploratory male single high-fidelity drug-induced arrhythmogenic risk classifier is shown in the right plot.

The subsequent training and development of the male multi-fidelity arrhythmogenic risk classifier is showcased in Figure 7. The upper panel plots showcase the evaluation of 10 high-fidelity evaluations of {β_CaL_, β_Kr_} on male drug-induced arrhythmogenesis. Left, the virtual electrocardiograms showcase the effect that various drug-induced ion channel blocking combinations have on the male heart. Here, only one exploratory sample (β_CaL_ = 6.7%, β_Kr_ = 92.6%) resulted in reentrant arrhythmia in the male heart. The other {β_CaL_, β_Kr_} combinations affected the QT interval, but did not lead to arrhythmogenesis. In the upper middle plot, the *N*_*L*_ = 50 low-fidelity evaluations are shown together with the first 10 exploratory high fidelity arrhythmogenicity classifications. On the upper right, the initial multi-fidelity Gaussian process risk classifier for male arrhythmogenesis is shown. Concomitantly, Figure A3 showcases a male single-fidelity risk classifier, only taking into account these high-fidelity arrhythmia development evaluations. Comparing the upper panel plots of Figure 7 with Figure A3, it can be seen that taking the low-fidelity classification data into account in training a Gaussian process classifier significantly aids the precision of the high-fidelity classifier with a limited amount of samples. This is the power of multi-fidelity Gaussian process classification. In the lower panel plots of Figure 7, we showcase the multiscale evaluation of 15 additional high-fidelity active learning samples. These samples allowed us to capture the bias in the low-fidelity predictions (see Equation 13) showcased in more detail in Figure A5 (left). The virtual electrocardiogram recordings of a subset of these active learning samples, four arrhythmic and four non-arrhythmic samples, are shown in the lower left and mid plots, respectively. Finally, the fully explored and exploited male multi-fidelity arrhythmogenic risk classifier is shown in the lower-right plot, with *N*_*L*_ = 50 and *N*_*H*_ = 25 low- and high-fidelity risk evaluations, respectively.

**Figure 8:**
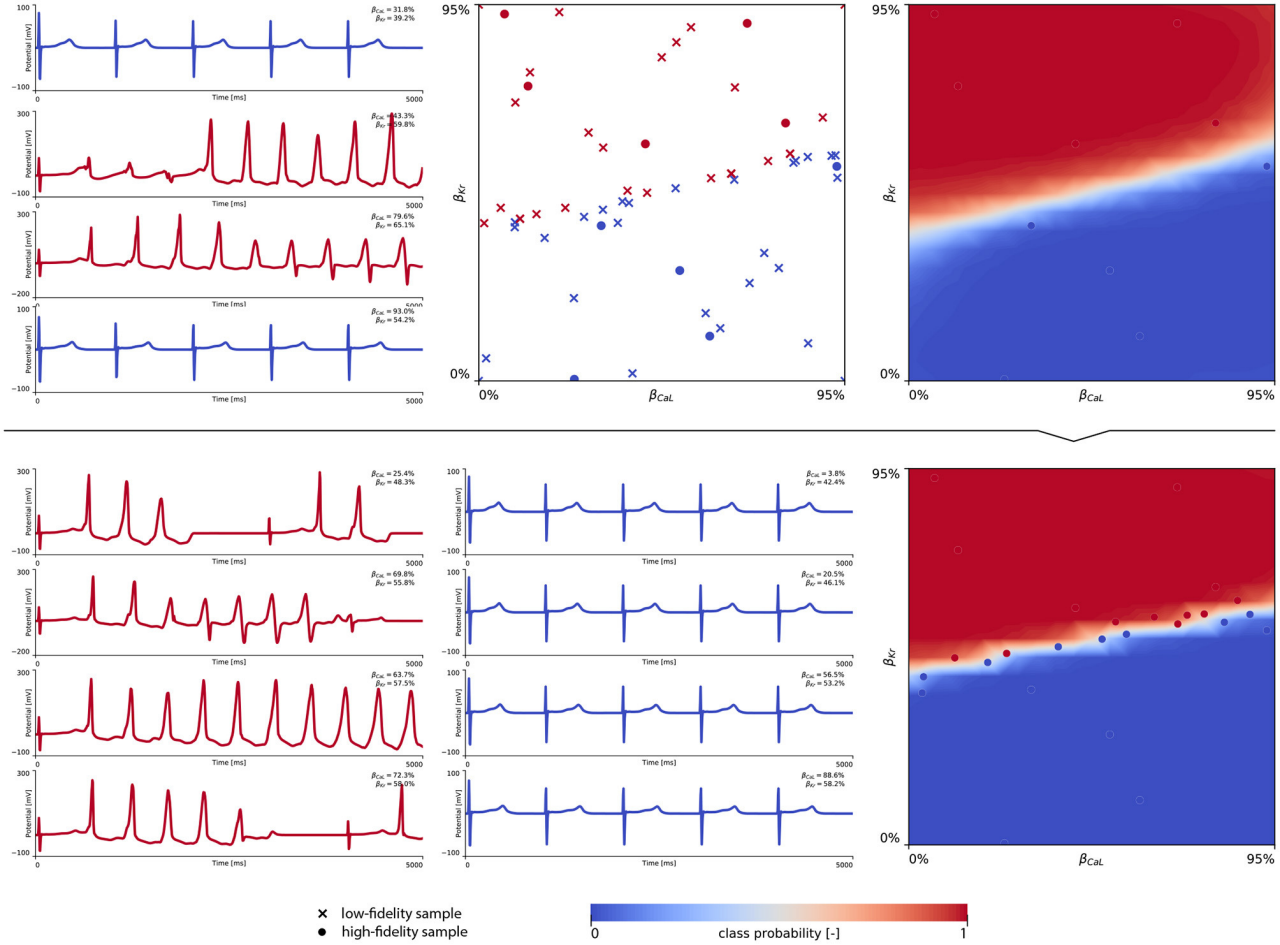
**Female multi-fidelity drug-induced arrhythmogenic risk classifier**. **(Upper)** Initial exploration of the female drug-induced arrhythmogenic risk parameter space. The first 10 internal Latin hypercube samples of the $B=\left\{\beta_{\text{Kr}},\beta_{\text{CaL}}\right\}$ parameter space were evaluated for female drug-induced arrhythmogenesis, as showcased in the upper middle plot with “dot” markers. Here, the low-fidelity samples from training the low-fidelity female midwall Gaussian process classifier are shown with “x” markers. Virtual electrocardiograms of two arrhythmic and two non-arrhythmic {β_Kr_, β_CaL_} samples are shown in the upper left column plots. The resulting drug-induced arrhythmogenicity probabilities for the female heart is shown in the upper right plot. **(Lower)** Active learning exploration and exploitation of the female drug-induced arrhythmogenic risk classification boundary. The multi-fidelity Gaussian process classifier was trained further using 15 additional active learning high-evaluations of {β_Kr_, β_CaL_} effects. On the lower left, four additional arrhythmic sample evaluations are shown. The lower middle plot showcases four additional non-arrhythmic sample evaluations. The final female multi-fidelity drug-induced arrhythmogenic risk classification boundary is shown in the lower right plot.

**Figure A4:**
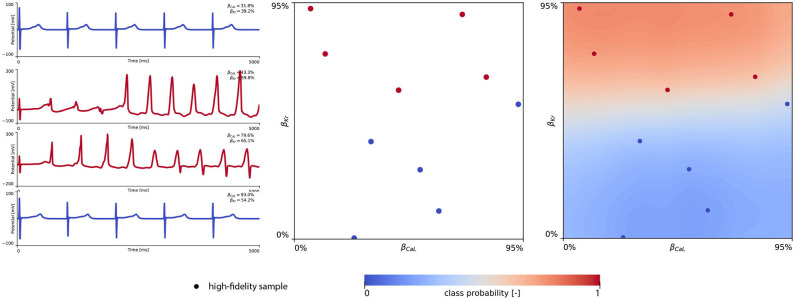
Female single-fidelity multiscale arrhythmogenic risk classifier. Initial single high-fidelity exploration of the female drug-induced arhhythmogenic risk parameter space. The middle plot showcases 10 internal latin hypercube {β_Kr_, β_CaL_} sample evaluations of arrhythmogenesis in the female heart. The virtual electrocardiograms for two arrhythmogenic and 2 non-arrhythmic sample evaluations are shown in the left plots. The resulting exploratory female single high-fidelity drug-induced arrhythmogenic risk classifier is shown in the right plot.

**Figure A5:**
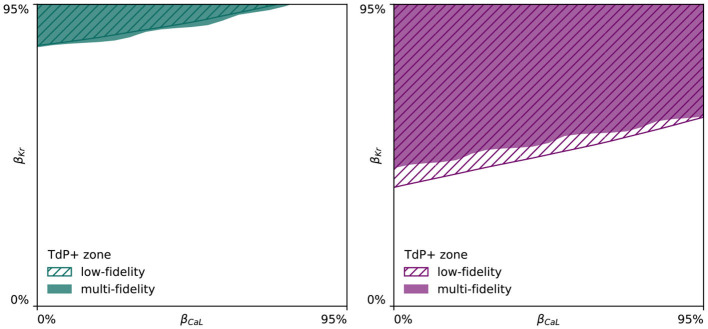
Low- vs. multi-fidelity arrhythmogenicity mismatch. Mismatch between the low-fidelity midwall cell proxy and the full high-fidelity multiscale simulations for arrhythmogenic risk classification. For the male arrhythmogenic risk classification **(Left)**, this mismatch is limited. For the female arrhythmogenicity classification **(Right)**, the multi-fidelity classifier predicts arrhythmogenesis at larger {*I*_Kr_, *I*_CaL_} channel blocking combinations than the low-fidelity risk classifier.

Figure 8 showcases the training of the female multi-fidelity arrhythmogenic risk classifier. In evaluating 10 high-fidelity Latin hypercube samples, five ion channel blocking samples drove the female heart to arrhythmogenesis, as shown in the upper middle plot. The electrocardiograms of two arrhythmic and two non-arrhythmic samples are shown in the upper left plots. The upper right plot depicts the initial female multi-fidelity drug-induced arrhythmogenicity classifier, taking into account all low-fidelity abnormality classification samples and the first 10 exploratory high-fidelity drug-induced arrhythmogenesis evaluations. Again, comparing this multi-fidelity classifier to the single-fidelity multiscale classifier shown in Figure A4 showcases the power of multi-fidelity Gaussian process risk classification. Through active learning, the low- to multi-fidelity bias (Equation 13) is inferred from 15 additional high-fidelity arrhythmogenic risk evaluations and depicted in Figure A5. Compared to the male heart, our results showcase a larger low- to multi-fidelity bias for the female heart. The lower left and mid plots in Figure 8 delineate the virtual electrocardiograms of four arrhythmogenic and four non-arrhythmogenic high-fidelity active learning sample evaluations, respectively. The final female multi-fidelity drug-induced arrhythmogenic risk classifier is shown in the lower-right plot.

Both male and female multi-fidelity drug-induced arrhythmogenicity classifiers highlight the pro-arrhythmic effect of *I*_Kr_ ion channel blocking and the anti-arrhythmic effect of *I*_CaL_ ion channel blocking. For the male heart, we predict drug-induced arrhythmogenicity at 81.7% *I*_Kr_ blocking when there is no *I*_CaL_ blocking. At 25.0, 50.0, and 75.0% *I*_CaL_ blocking, the critical β_Kr_ is 83.8, 88.6, and 94.6%, respectively. For 100% *I*_CaL_ blocking, no arrhythmia develops, regardless of the amount of *I*_Kr_ blocking. For the female heart, our risk classifier predicts drug-induced arrhythmogenesis at 43.7% blocking without any *I*_CaL_ blocking. For 25.0, 50.0, 75.0, and 100% *I*_CaL_ blocking, arrhythmia develops at 48.1, 52.2, 56.5, and 59.9% *I*_Kr_ blocking, respectively. Overall, the female heart can be expected to be significantly more prone to drug-induced arrhythmogenicity.

### 3.4. Sex-Specific Drug Risk Stratification

**Figure 9:**
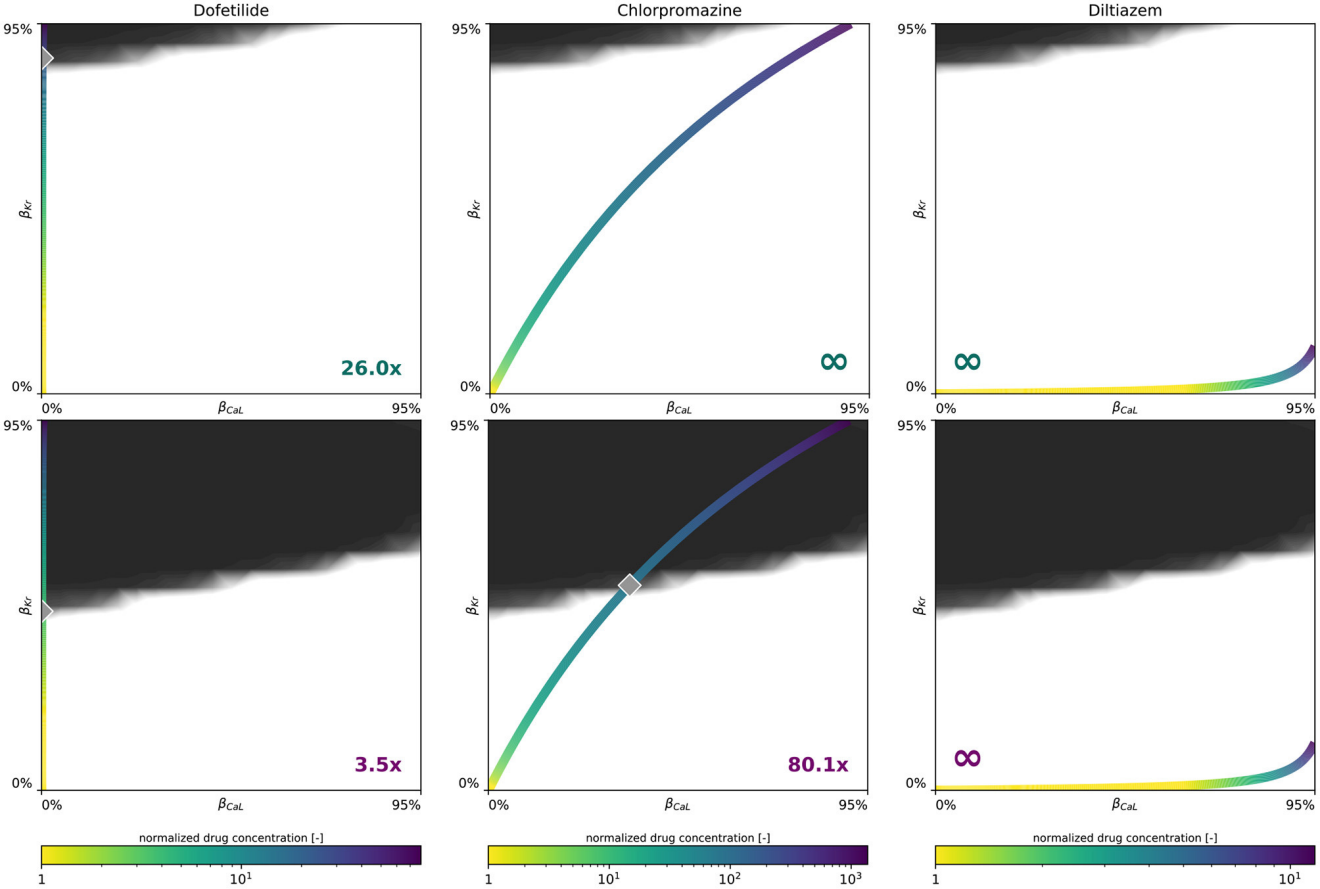
**Sex-specific drug-induced arrhythmogenic risk assessment**. Male **(Upper)** and female **(Lower)** drug-induced arrhythmogenic risk assessment for dofetilide, a high risk drug, chlorpromazine, an intermediate risk drug, and diltiazem, a low risk drug. The color-coded line represents drug-specific concentration-dependent *I*_CaL_/*I*_Kr_ ion channel blocking trajectory, normalized with respect to the drug's effective free therapeutic plasma concentration. The gray diamond shaped marker highlights the critical concentration, annotated in the plot's bottom corner, at which the drug's block-concentration trajectory crosses the mean multi-fidelity arrhythmogenicity classification boundary.

Figure 9 demonstrates how we use our male and female multifidelity arrhythmogenicity classification boundary to perform a sex-specific drug risk assessment. More specifically, we calculate the drug-induced arrhythmogenic risk for dofetilide, a high risk drug, chlorpromazine, an intermediate risk drug, and diltiazem, a low risk drug. For each of these drugs, the drug-specific color-coded block-concentration characteristics map onto a trajectory in the β_CaL_/β_Kr_ plane. The intersection of this trajectory with our trained classification boundary defines the critical drug concentration at which arrhythmia can be expected to develop. For dofetilide, the block-concentration curve crosses the male and female arrhythmogenicity classification boundary at 26.0x and 3.5x the drug's effective free therapeutic plasma concentration, respectively. For chlorpromazine, the block-concentration curve does not cross the male classification boundary, signifying this drug can be considered safe for men. For women, the chlorpromazine block-concentration and classification boundary intersect at a critical concentration of 80.1x. For diltiazem, the block-concentration trajectory does not cross the male, nor the female arrhythmogenicity classification boundary, showcasing this drug to be safe for both sexes.

## 4. Discussion

### 4.1. Our Motivation for Multiscale Modeling

Until recently, the gold standard to assess pharmacological pro-arrhythmic risk consisted of assessing the potential of a drug (1) to cause pharmacological block of the rapid-delayed rectifier potassium *I*_Kr_ current encoded by the human ether-à-gogo related gene and (2) to prolong the QT interval in electrocardiographic animal and human studies. Although these biomarkers show good sensitivity, they are costly and have poor specificity, potentially blocking safe new drugs from ever reaching the market (Sager, 2008). In response to this problem, the Comprehensive *in vitro* Proarrhythmia initiative was launched (Sager et al., 2014). This incentive aimed to develop novel drug-induced arrhythmia biomarkers through a combined *in vitro* and *in silico* approach. *in vitro*, insights into the effect of drugs on multiple ion channels in the cardiomyocyte were collected. *In silico*, these insights were used to develop a mechanistic understanding how these ion channel blockings affect cardiac electrophysiology and function. Over the past decade, our *in silico* mechanistic understanding of the effects of drugs on cardiac electrophysiology has taken big leaps forward. As such, drug cardiotoxicity has been extensively studied in ventricular myocyte models (Mirams et al., 2011; Passini et al., 2017), transmural cable simulations (Moreno et al., 2013; Romero et al., 2018), planar and cubic tissue slabs (Kubo et al., 2017; Yang et al., 2020; Margara et al., 2021), and even ultra-high resolution, multiscale heart models (Wilhelms et al., 2012; Okada et al., 2015; Sahli Costabal et al., 2018a; Hwang et al., 2019). Within this paradigm, multiple groups have used such models to develop arrhythmogenic risk classifiers. These *in silico* augmented biomarkers showcase improved sensitivity and specificity with respect to the gold standard human ether-à-gogo and QT prolongation guidelines (Passini et al., 2017; Li et al., 2019). Currently, these *in silico* arrhythmogenicity biomarkers focus mainly on lower-fidelity isolated cardiac cell models (Lancaster and Sobie, 2016; Britton et al., 2017; Fogli Iseppe et al., 2021) or simplified cable simulations (Polak et al., 2018; Romero et al., 2018; Yang et al., 2020). The underlying motivation for such an approach is the role of cellular early afterdepolarizations and repolarization failures in providing a trigger for the development of arrhythmia. Nevertheless, arrhythmogenicity is not completely governed by, nor exclusively limited to, depolarization or repolarization abnormalities (Pugsley et al., 2015). Overall, the spatial dispersion of repolarization within the ventricular myocardium has been identified as the principal arrhythmogenic substrate (Antzelevitch and Burashnikov, 2011). A recent computational multiscale cardiac electrophysiology study showcased that the electrotonic coupling effect in tissue is an essential factor to predict drug effects on the living organ (Kubo et al., 2017). More specifically, computational 2D tissue slab results revealed no tachyarrhythmia in the presence of early afterdepolarizations at the cellular level, and arrhythmogenic induction in between the endocardial and midwall tissue layers, rather than in the midwall tissue itself. As such, it can be appreciated that accurate mechanistic understanding of arrhythmogenesis requires a high-fidelity multiscale modeling approach coupling the effect of drugs to subcellular ion channel activity, to cell-to-cell coupling at the tissue scale, and the tissue's three-dimensional heterogeneous and anisotropic organization at the organ scale.

### 4.2. Our Motivation for Multi-Fidelity Gaussian Process Classification

The current lack of multiscale computional modeling in the development of novel *in silico* augmented arrhythmogenic risk classifiers can be associated to their computational cost. Whereas a single cell action potential takes seconds to compute on a single CPU, a coupled cell-tissue-organ scale exposure-response simulator can easily take multiple hours to compute on a high performance computing cluster (Towns et al., 2014; Sahli Costabal et al., 2018a). Upon developing a risk classifier that evaluates the arrhythmogenic susceptibility to a whole series of drugs at multiple drug concentrations, the computational burden of performing a multiscale evaluation for each case becomes too high. To overcome this limitation, our study took a different approach. Instead of evaluating the case-by-case drug- and concentration-specific response, we trained a risk classifier based on the most important drug-induced ion channel blockings. We used a combined multiscale modeling and machine learning approach, entailing logistic regression, single- and multi-fidelity Gaussian process classification, and active learning techniques. First, we quantified the principal role that drug-affected ion channel currents play in developing arrhythmia. Using these insights, we established a two-dimensional drug blocking parameter space in which we evaluated the arrhythmogenic susceptibility of various drug-induced ion channel blocking combinations. Next, we relied on Gaussian process classification to delineate the arrhythmogenicity border within the considered parameter space. Given the high computational cost of each multiscale evaluation, we set up a multi-fidelity framework. Here, we used cellular midwall cell evaluations as a low-fidelity proxy for arrhythmogenic risk classification. This low-fidelity classifier was subsequently used to inform the underlying shape of the multi-fidelity classifier. Doing so, we minimize the amount of high-fidelity evaluations within the studied parameter space, and still end up with a precise multiscale arrhythmogenic risk classification boundary. This multi-fidelity ion channel blocking classifier subsequently allows us to post-process the intrinsic arrhythmogenic risk for each possible drug and concentration at no additional computational cost, without losing accuracy of the underlying multiscale arrhythmic and non-arrhythmic classification. We took advantage of the probabilistic nature of our Gaussian process classifiers to implement an effective data acquisition via active learning strategies. These strategies sought a balance between parameter space exploration and classification boundary exploitation. Consequently, our methodology allowed us to maximized classifier accuracy under a constrained computational budget and provided a significant advantage over other classifiers including logistic regression and support vector machines.

### 4.3. Our Motivation for Studying Sex Differences

About a century ago, sex differences in cardiac electrophysiology were reported for the first time (Bazett, 1920). Throughout the past two decades, these insights have matured into the recognition that female sex is an essential risk factor for multiple adverse cardiac events (Yarnoz and Curtis, 2008). Especially for drug-induced arrhythmogenesis, women turn out to be impacted twice as much as men (Makkar, 1993; James et al., 2007; Coker, 2008). Nevertheless, the effect of sex differences on cardiac electrophysiology and drug-induced arrhythmogenicity remain largely underexplored. With current sex-agnostic population-based models (Muszkiewicz et al., 2016; Li et al., 2019) being calibrated on *in-vitro* studies, which tend to be male-dominated (Ramirez et al., 2017), sex bias is expected to propagate through these novel *in silico* augmented arrhythmogenic risk classifiers. This study sought to take female sex into account as an independent biological variable by developing two sex-specific *in silico* augmented multiscale arrhythmogenic risk classifiers. To accomplish this, we first extended the multiscale envelope of studying sex-differences in cardiac electrophysiology beyond the cell or tissue level (Yang et al., 2017; Fogli Iseppe et al., 2021) up to the organ scale. Next, we used the developed framework to delineate male vs. female arrhythmogenic sensitivity to drugs.

### 4.4. Male vs. Female Cardiac Electrophysiology Across the Scales

Our male and female cell models were based on a high-throughput quantitative assessment of genome-scale sex differences in male and female human endo- and epicardial tissue (Gaborit et al., 2010). The resulting female endo- and epicardial action potential duration is significantly longer than the male action potential durations. Both the male and female endo- and epicardial action potential durations in this study are smaller than those computed in other studies (Yang and Clancy, 2012). Whereas other studies considered the baseline O'Hara-Rudy model and ion channel conductances to form the male baseline cell model (Yang and Clancy, 2012), our approach acknowledged the originally reported data population (O'Hara et al., 2011) and regarded the baseline model as a mixed 56% male / 44% female generalized model. Despite these differing views, our computed action potential durations fall well within the reported ranges based on experimental variability (Gaborit et al., 2010; Yang and Clancy, 2012). Similarly, the range of our sex-specific endo- and epicardial action potential durations are in agreement with reported populations of ventricular cell models (Britton et al., 2017). Our midwall cell action potential durations also fall within the same reported population variability. Averaged over the three cell types, our simulated female cells take 30% longer to repolarize than their male counterparts, which is consistent with the reported 29% relative female-to-male action potential differences for human ventricular myocytes (Verkerk et al., 2005). Focusing on the restitution behavior, our reported male and female action potential durations at 90% repolarization in Figure 3 agree favorably with previously reported experimental data for human tissue (Morgan et al., 1992; Drouin et al., 1995; Li et al., 1998; ten Tusscher et al., 2004; O'Hara et al., 2011).

At the organ scale, the combined effect of sex-specific cell-scale ion channel activity, tissue-scale conductivity and organ-scale geometry results in a shorter QRS and longer QT interval for women. Both results are in agreement with clinical population studies. Female vs. male QRS shortening of 5 ms, vs. 4 ms here, and a QT prolongation of 20 ms, vs. 63 ms here, have been reported in the literature (Vicente et al., 2014). The mismatch between a recorded 29% AP prolongation and a clinical QT prolongation of ‘only’ 2-6% has been hypothesized to be related to the mismatch between single isolated cell behavior and three-dimensional electrophysiologically coupled heterogeneous tissue (Verkerk et al., 2005). Indeed, our multiscale models showcase that a 30% action potential prolongation between both sexes at the cell scale only resulted in a male QT interval which was 15% shorter than the female QT interval at the organ scale. Nevertheless, this sex difference in QT interval duration is still on the higher end. This discrepancy seems to be related to our male multiscale heart model. The computed male QT interval of 348 ms corresponds to the 5th percentile of the clinically reported ranges for men (Asatryan et al., 2021), whereas our computed female QT interval of 411 ms aligns perfectly with the clinically reported range of 386–445 ms (Vicente et al., 2014). As our multiscale models demonstrated the dominant role that changes in ion channel activity have on the timing of the T wave end, there is a strong need for an in-depth experimental study on the sex-specific differences in functional ion channel activity of non-diseased human ventricular myocytes. Unfortunately, we are not aware of such data being currently available. Similarly, studies have shown that the inclusion of interventricular and apicobasal ion channel gradients at the tissue scale can further impact ECG morphology (Okada et al., 2011). Emerging electrocardiographic imaging techniques show great potential to study sex differences in healthy tissue-scale conductivity in more detail but remain challenging (Cluitmans et al., 2018; Andrews et al., 2019).

### 4.5. A Novel Multiscale Sex-Specific Arrhythmogenic Risk Classification

Given the high amount of ionic currents constituting the electrophysiological behavior of human ventricular cardiomyocytes (Equation 4), studying the drug-induced risk to develop arrhythmia requires the exploration of a large parameter space constituting different amounts of drug-induced blocking of each and every possible ion channel. To keep the parameter space computationally tractable, we focused on the seven most important ion channels for arrhythmogenic risk stratification, and used logistic regression to quantify their relative importance. The normalized marginal effects of drug-induced ion channel blocking on arrhythmic sensitivity in Figure 6 identify β_Kr_ and β_CaL_ as the key pro-arrhythmic and anti-arrhythmic ion channel blockings, respectively. This conclusion is consistent with previous sex-agnostic risk analyses (Crumb et al., 2016), and is thus found to hold true across men and women. Interestingly, our analysis highlights a relatively decreased protective role of L-type *Ca*^2+^ channel blocking in women. The higher amount of recorded de- and repolarization abnormalities confirmed the higher susceptibility of female cardiomyocytes to drug-induced arrhythmogenicity. These results agree well with experimental exploratory studies on cell-scale sex differences in drug-induced arrhythmogenicity (Liu et al., 1999; Verkerk et al., 2005). The male and female multi-fidelity arrhythmogenic risk classifiers in Figures 7, 8, respectively quantify this differing risk with increased fidelity, as shown in Figure A5. Overall, we found the female heart to demonstrate arrhythmogenicity at lower drug-induced *I*_Kr_ and *I*_CaL_ ion channel blocking than the male heart. Interestingly, our previous work on sex-agnostic arrhythmia risk assessment in the heart showcased an arrhythmogenic risk classification boundary in between the male and female arrhythmia risk classification boundary developed in this study (Sahli Costabal et al., 2019c). As such, we conclude that a generalized sex-agnostic arrhythmia risk classification underestimates and overestimates the cardiac toxicity of drugs for women and men, respectively. This directly puts women at higher risk for drug-induced arrhythmogenicity events, explaining the higher incidence reported in women (Makkar, 1993; James et al., 2007; Coker, 2008).

In applying our novel sex-specific arrhythmogenic risk classifier to a high, intermediate and low risk drug, we quantify this increased risk for women in more detail. For dofetilide, a class III anti-arrhythmic agent, both the male and female arrhythmia risk classifier confirm the general notion that dofetilide can have dramatic consequences if not dosed correctly (Briceño and Supple, 2017). For men, our risk classifier predicts a spontaneous transition from a sharp but smoothly propagating excitation pattern into rapid, irregular, asynchronous activation patterns at a critical concentration of 26.0x. For women, the same risk is predicted at 3.5 times the drug's free therapeutic plasma concentration. These results agree well with clinical trials where female sex was associated with three-fold higher odds of dofetilide discontinuations or dose reductions relative to the male sex (Pokorney et al., 2018). Most dosage reductions led to half of the recommended dosage for women. Interestingly, women were highly underrepresented in original clinical trials assessing the safety of dofetilide, only accounting for 28 and 16% of the total amount of enrolled patients (Køber et al., 2000; Singh et al., 2000). For chlorpromazine, an antipsychotic drug, our female arrhythmogenic risk classifier estimated a risk for arrhythmogenesis at 80.1x concentration, whilst for men no arrhythmogenicity was predicted. As expected from such a high critical risk concentration, chlorpromazine-induced arrhythmogenicity can be expected to be uncommon. Indeed, a comprehensive literature search spanning four decades of clinical case report data identified only seven published cases of chlorpromazine-associated ventricular arrhythmia. All these cases involved women (Hoehns et al., 2001). Finally, for diltiazem, a calcium channel blocker used to manage blood pressure and chest pain, the drug's concentration-block trajectory does not cross our male nor female multi-fidelity arrhythmogenic risk classification boundary. Consequently, we predict no arrhythmogenesis for diltiazem and consider this drug to be safe, both for men and women. This risk assessment corresponds well with diltiazem's ‘low/no arrhythmia risk’ classification by a team of clinical cardiologists and electrophysiologists based on publicly available data and expert opinion (Colatsky et al., 2016). Additionally, the arrhythmogenic safety of diltiazem was also confirmed by recent sex-agnostic population-based arrhythmia risk classifiers from other research groups (Lancaster and Sobie, 2016; Li et al., 2019). Importantly, these sex-specific drug-induced arrhythmogenic risk assessments assume no other medications to be taken concomitantly with these drugs.

In this study, we first build a multiscale mechanistic understanding of arrhythmogenesis in the male and female heart, and subsequently use these computational insights to evaluate the sex-specific drug-induced arrhythmogenic risk. This approach is inherently different from a recent first approach toward sex-specific drug-induced arrhythmogenicity classification (Fogli Iseppe et al., 2021). In this study, the authors focus on the *in silico* computed effect of drugs on male and female human epicardial cardiomyocytes. Using statistical learning techniques, they identify the key synthetic action potential biomarkers contributing to the most accurate prediction of arrhythmogenicity outcomes for men and women specifically. This approach relied on a ground truth classification assumption that risky drugs are dangerous for men and women, and safe drugs are safe for both men and women. These classifications were deduced from reported adverse event analyses performed within the Adverse Drug Event Causality Analysis (Woosley et al., 2017). With female sex reported to be historically highly underrepresented in clinical studies (Vitale et al., 2017), such an assumption could potentially be problematic, especially for older drugs. Our study offers the benefit of using a full multiscale framework to inform arrhythmogenic risk from a mechanistic understanding. Apart from this differing approach to arrhythmogenic risk classifier development, our study also takes into account the effect of midwall cells in an individual's predisposition to arrhythmogenesis (Drouin et al., 1995; O'Hara et al., 2011) and did not assume the baseline O'Hara-Rudy model to represent a purely male endocardial cell model, as discussed in section 2.2.1. Consequently, these differing approaches render a one-on-one comparison between our studies difficult. Nonetheless, for chlorpromazine, the only drug that was studied in both studies, both our studies classify this drug safe for men and at medium risk for women. Based on our sex-specific arrhythmia risk classifiers in which the male arrhythmogenic {β_CaL_, β_Kr_} risk zone ⊂ the female arrhythmogenic {β_CaL_, β_Kr_} risk zone, our classifiers do not predict any drug to have a higher risk for women than for men. Interestingly though, the alternative approach identified specific drugs that are safer for women than for men (Fogli Iseppe et al., 2021). This disagreement might be associated with the current ambiguity on functional sex differences in Ca^2+^ handling (Parks and Howlett, 2013; Parks et al., 2014) which led the authors to disregard the genomic sex differences in sodium potassium *I*_NaK_ pump and Ca^2+^ uptake channel activities and relatively upscale the female sodium calcium exchange currents we deduced in Table 1. Currently, experimental data on Ca^2+^ in healthy human myocardium are lacking, and further investigation on these functional sex differences is warranted to improve our sex-specific arrhythmogenic risk classifiers in the future. Additionally, limiting our risk classifier to only take β_CaL_ and β_Kr_ into account might not uncover this behavior. It can for example be seen from Figure 6 that β_NaL_ has a stronger anti-arrhythmic effect for female midwall cardiomyocytes than it does for men, and thus including this feature as a third drug-induced ion channel blocking input feature to our arrhythmia risk classifier might lead to male and female three-dimensional arrhythmogenic risk zones that do not completely overlap. Apart from these discrepancies, the overall conclusion is the same: including sex as a new independent factor in preclinical cardiotoxicity risk assessment is crucial to avoid potentially life-threatening consequences for the female population (Chorin et al., 2017). Given the absence of reliable large-scale arrhythmogenic risk assessments for women specifically, and the male dominance in clinical studies, our study forms an important first step toward mechanistically uncovering the role that sex differences on the subcellular, cellular, tissue, and organ scale play in drug-induced arrhythmogenicity. An improved understanding of these sex-specific mechanisms will be crucial to provide new therapeutic approaches that do no longer put women at increased risk.

### 4.6. Limitations and Outlook

Although our proposed methodology holds great promise to rapidly assess the sex-specific risk of a new drug, without relying on clinically reported adverse event occurrence, it has a few limitations: first, our sex-specific multiscale exposure-response simulators are only as good as their input. In the long term, more sex-specific human cell and tissue experiments are needed to fine-tune the cell- and tissue-scale sex differences in ion channel activity and conductivity currently deduced from genomic data. Such experimental data would also be highly desirable to resolve the current debate on the existence or non-existence of sex differences in Ca^2+^ handling. The lacking human experimental data for more in-depth sex-specific validation of our multiscale simulation outcomes suggest important avenues for further studies. Novel developments of male and female hIPSC-derived cell lines might provide an interesting route to study this further (Huo et al., 2019). Second, our developed risk classifiers currently focused on the risk of drug-induced *I*_Kr_ and *I*_CaL_ blocking. Even though we identified these ion channel blockings to be the most critical channels for drug-induced arrhythmogenesis for both the male and female heart, arrythmogenic risk stratification for drugs that mainly target other channels might require including additional ion channel blockings to our risk classifiers. As our results in Figure 6 showcase, extending both the male and female risk classifiers to take into account drug-induced *I*_Ks_ and *I*_NaL_ blocking would be the most logical next step. Third, given the role that the excitation rate has on the electrophysiological behavior of the human heart, we aim to extend our classifiers to take into account heart rate in our future work. Fourth, at this point, we did not take into account male and female population variability. As additional experimental data becomes available, more in-depth sex-specific population studies form an interesting next step. We have developed efficient frameworks to quantify and propagate such uncertainty through computational models in the past (Peirlinck et al., 2019; Sahli Costabal et al., 2019a), and look forward to apply these techniques to this problem. Prior to this, a critical and logical next step would be to validate our method using our own independent experiments with human adult cardiomyocytes, and ideally, healthy human volunteers. Ultimately, with a view toward precision cardiology, this sex-specific approach forms an important initial step toward identifying the optimal course of care for each individual patient based on personalized block-concentration characteristics and personalized cardiac heart models (Trayanova, 2018; Peirlinck et al., 2021; Rodero et al., 2021).

## 5. Conclusion

The objective of this study was to quantify sex differences in drug-induced arrhythmogenesis. Toward this goal, we created sex-specific male and female multiscale exposure-response simulators. These simulators differ in subcellular ion channel activity, tissue-level conductivity, and organ-scale geometry. Using logistic regression, we identified the rapid delayed rectifier potassium channel *I*_Kr_ and the L-type calcium channel *I*_CaL_ as the most importance ion channels to modulate male and female arrhythmogenesis on the cellular level. Based on these findings, we created an exploratory ion channel block parameter space and combined low-fidelity cell-scale and high-fidelity multiscale modeling to delineate arrhythmogenic risk classification boundaries. Our study quantitatively confirms and delineates women's intrinsically higher risk for drug-induced arrhythmia both on the cell and organ scales. We applied our new sex-specific multi-fidelity pharmacological risk classifiers to assess critical drug concentrations for a high, an intermediate, and a low risk drug. For the high risk drug dofetilide, our predicted critical drug concentration for female hearts is seven times lower than for male hearts. This result explains, at least in part, why women are more likely than men to develop drug-induced arrhythmia. Acknowledging and understanding sex differences in drug safety evaluation is critical when developing new drugs and prescribing existing drugs in combination with other drugs.

## Data Availability Statement

The original contributions presented in the study are included in the article, further inquiries can be directed to the corresponding authors.

## Author Contributions

MP was responsible for conception and design of the study, data analysis and interpretation, and draft of the manuscript. FS and EK contributed to and guided study conception and design and provided critical revision of the manuscript for intellectual content. All authors approved the final version of the article to be published.

## Conflict of Interest

The authors declare that the research was conducted in the absence of any commercial or financial relationships that could be construed as a potential conflict of interest.

## Publisher's Note

All claims expressed in this article are solely those of the authors and do not necessarily represent those of their affiliated organizations, or those of the publisher, the editors and the reviewers. Any product that may be evaluated in this article, or claim that may be made by its manufacturer, is not guaranteed or endorsed by the publisher.

## A. Appendix

### A.1. Single-Fidelity Cell-Scale Risk Classifiers

Figures A1, A2 showcase the exploration and exploitation progress in training a single-fidelity Gaussian process classifier for drug-induced de- and repolarization abnormalities in male and female midwall cardiomyocytes respectively. In the upper middle plots, we summarize the binary risk classifications in 25 latin hypercube samples of the $B=\left\{\beta_{\text{Kr}},\beta_{\text{CaL}}\right\}$ using Myokit. We highlight two exploratory {β_Kr_, β_CaL_} sample evaluations from each class in the upper left plot. Using this initial B classification dataset, we trained the initial risk classifier shown in the upper right plot. Subsequently, the risk classification boundary was further explored and exploited using active learning. By actively sampling new {β_Kr_, β_CaL_} samples with high variance in the posterior Gaussian Process classification distribution close to the classification boundary, we cost-effectively enhance the accuracy of our classification boundary. We showcase four active learning samples for which we recorded de- or repolation abnormalities in the lower left plots, and four active learning samples for which no abnormalities were recorded in the lower middle plots. With a total of 25 additional active learning samples, we produced the cellular male and female single-fidelity risk classifier displayed in the lower right plots of Figures A1, A2 respectively. Comparing the initial and final risk classifier, we showcase the power of active learning to improve classification confidence.

### A.2. Single-Fidelity Multiscale Arrhythmogenic Risk Classifiers

Figure A3 showcases the exploration of male drug-induced arrhythmogenic risk classification based on 10 high-fidelity evaluations of the $B=\left\{\beta_{\text{Kr}},\beta_{\text{CaL}}\right\}$ parameter space. By training a single-fidelity Gaussian process classifier with a dataset that comprised one arrhythmogenic and nine non-arrhythmic {β_Kr_, β_CaL_} samples (see Figure A3 - left and mid), the resulting exploratory Gaussian process risk classifier (Figure A3 - right) predicts a low probability for drug-induced arrhythmogenesis. It can be expected that a significant amount of additional computationally expensive high-fidelity sample evaluations would be needed to accurately detect the *y*^*H*^ = 1 region (Sahli Costabal et al., 2019b). If we compare this exploratory single-fidelity arrhythmogenic risk classifier to the exploratory multi-fidelity arrhythmia risk classifier shown in the upper panels of Figure 7, it can be seen that the multi-fidelity classifiers greatly benefits from the candidate boundary encoded in the low-fidelity data. As such, for the same amount of high-fidelity evaluations, the exploratory multi-fidelity arrhythmogenic risk classifier in Figure 7 showcases a significantly higher precision and accuracy than the exploratory single-fidelity arrhythmia risk classifier in Figure A3. This power of multi-fidelity risk classification, opposed to single-fidelity risk classification, can also be appreciated in Figure A4. Even though the initial exploratory dataset consisted of five arrhythmogenic and five non-arrhythmic samples (see Figure A4 - left and mid), the resulting single-fidelity risk classifier (Figure A4 - right) provides lower classification confidence than the exploratory multi-fidelity risk classifier in Figure 8 (top).

### A.3. Low- Versus Multi-Fidelity Arrhythmogenicity Bias

Figure A5 highlights the recorded mismatch between the use of a low-fidelity midwall cell proxy for arrhythmogenic risk classification and a multi-fidelity risk classifier taking into account high-fidelity multiscale evaluations of the drug effect on the transmembrane potential evolution at the cell, tissue, and organ scale.

It can be seen that for the male arrhythmogenicity classifier, this mismatch is rather limited. However, for the female arrhythmogenic risk classification boundaries, the low-fidelity risk classification boundary predicts arrhythmogenesis at lower drug-induced {*I*_Kr_, *I*_CaL_} blocking combinations. This mismatch showcases the importance of taking the effect of electrotonic coupling and repolarization dispersion across the three-dimensional heterogeneous tissue organization into account.

**Table A1:** Sex-based differences in ion channel subunit expression from non-diseased human ventricles.

**Ion channel**	**Gene/protein**	**Epi**		**Endo**			**Mid**	
		**male**	**female**	**male**	**female**	**interp**	**male**	**female**
*I*_NaL_	Na_V_1.5	310.6	260.8	426.8	373.8	403.5		
	Ratio	0.77	0.65	1.06	0.93		1.06	0.93
		Based on ΔNa_V_1.5 expression (O'Hara et al., 2011).						
*I*_pCa_	Ca^2+^ ATPase 4	377.0	682.1	426.8	685.2	540.5		
	Ratio	0.70	1.26	0.79	1.27		1.97	3.17
		Based on ΔCa^2+^ ATPase 4 expression (Yang and Clancy, 2012).						
*I*_Kr_	hERG	179.5	144.2	164.8	130.5	149.7		
	Ratio	1.20	0.96	1.10	0.87		0.88	0.70
		Based on ΔhERG expression (O'Hara et al., 2011).						
*I*_Ks_	MinK	13.6	7.3	11.9	5.8	9.2		
	Ratio	1.16	0.93	1.10	0.88		1.10	0.88
		Based on 1/3 Δ MinK expression (Yang and Clancy, 2012).						
*I*_K1_	Kir2.3	91.2	21.4	92.7	55.2	76.2		
	Ratio	1.07	0.76	1.07	0.91		1.39	1.18
		Based on 1/3 Δ Kir2.3 expression (Yang and Clancy, 2012).						
*I*_NaCa, i/ss_	NCX1	821.1	801.4	754.5	739	747.7		
	Ratio	1.10	1.07	1.01	0.99		1.41	1.38
		Based on Δ NCX1 expression (O'Hara et al., 2011).						
*I*_NaK_	Na^+^/K^+^ ATPase α1	207.7	513.4	269	622.5	424.54		
	Na^+^/K^+^ ATPase α3	1481	917.8	1547.6	1014.2	1312.904		
	Ratio	0.92	0.87	1.00	1.00		0.70	0.70
		Based on 1/3 ΔNa^+^/K^+^ ATPase α1 and 2/3 ΔNa^+^/K^+^ ATPase α3 expression (Yang and Clancy, 2012).						
*I*_Kb_	KV1.5	12.7	6.5	19.5	10.5	15.54		
	Ratio	0.82	0.42	1.25	0.68		1.25	0.68
		Based on ΔK_V_1.5 expression (O'Hara et al., 2011).						
Ca^2+^ release	RYR2	6213.7	4890.6	5463.9	5582.5	5516.084		
	Ratio	1.13	0.89	0.99	1.01		1.68	1.72
		Based on ΔRYR2 expression (O'Hara et al., 2011).						
Ca^2+^ uptake	SERCA2	4850.5	6728.4	3410.4	3921.9	3635.46		
	Ratio	1.33	1.85	0.94	1.08		0.94	1.08
		Based on ΔSERCA2 expression (Yang and Clancy, 2012).						
[CMDN]	CALM3	1326.9	1955.5	1206.9	1600.5	1380.084		
	Ratio	0.97	1.28	0.92	1.11		0.92	1.11
		Based on 2/3 Δ CALM3 expression (Yang and Clancy, 2012).						

*Overview of the sex differences in ion channel subunit expression for the channels used in our cell-scale and multi-scale computational models. The gene/protein data represents the normalized relative expression (2-ΔCt) deduced from Gaborit et al. (2010). The endocardial interp column represents the relative ion channel subunit expression for the hypothesized 56% male, 44% female model that the original endocardial O'Hara Rudy cell model was based on. To compute the individual ion channel activity scalings, we followed scaling rules established by O'Hara et al. (2011) and Yang and Clancy (2012), as reported below each set of scaling ratios*.
